# An Ultrasensitive and Broad‐Spectrum MoS_2_ Photodetector with Extrinsic Response Using Surrounding Homojunction

**DOI:** 10.1002/advs.202408299

**Published:** 2024-10-16

**Authors:** Xiaoyan Liu, Jiaqi Zhu, Yufeng Shan, Changlong Liu, Changyi Pan, Tianning Zhang, Chixian Liu, Tianye Chen, Jingwei Ling, Junli Duan, Feng Qiu, Saqib Rahman, Huiyong Deng, Ning Dai

**Affiliations:** ^1^ School of Physics and Optoelectronic Engineering Hangzhou Institute for Advanced Study University of Chinese Academy of Sciences Hangzhou Zhejiang 310024 China; ^2^ Shanghai Institute of Optics and Fine Mechanics Chinese Academy of Sciences Shanghai 201800 China; ^3^ University of Chinese Academy of Sciences Beijing 100049 China; ^4^ State Key Labratory of Infrared Physics Shanghai Institute of Technical Physics Chinese Academy of Sciences Shanghai 200083 China; ^5^ Jiangsu Collaborative Innovation Center of Photovoltaic Science and Engineering Changzhou 213164 China

**Keywords:** broad‐spectrum photodetectors, extrinsic response, MoS_2_, N_2_ plasma implantation, surrounding homojunctions

## Abstract

As unique building blocks for advancing optoelectronics, 2D semiconducting transition metal dichalcogenides have garnered significant attention. However, most previously reported MoS_2_ photodetectors respond only to visible light with limited absorption, resulting in a narrow spectral response and low sensitivity. Here, a surrounding homojunction MoS_2_ photodetector featuring localized p‐type nitrogen plasma doping on the surface of n‐type MoS_2_ while preserving a high‐mobility underlying channel for rapid carrier transport is engineered. The establishment of p‐n homojunction facilitates the efficient separation of photogenerated carriers, thereby boosting the device's intrinsic detection performance. The resulting photoresponsivity is 6.94 × 10^4^ A W^−1^ and specific detectivity is 1.21 × 10^14^ Jones @ 638 nm, with an optimal light on/off ratio of ≈10^7^ at *V*_GS_ = −27 V. Notably, the introduction of additional bands within MoS_2_ bandgap through nitrogen doping leads to an extrinsic broadband response to short‐wave infrared. The device exhibits a photoresponsivity of 34 A W^−1^ and a specific detectivity of up to 5.92 × 10^10^ Jones @ 1550 nm. Furthermore, the high‐performance broadband response is further demonstrated through imaging and integration with waveguides, paving the way for next generation of multifunctional imaging systems and high‐performance photonic chips.

## Graphical abstract

A surrounding homojunction MoS_2_ photodetector featuring a localized p‐type nitrogen plasma doping region and an underlying high‐mobility channel is engineered. This design boosts the efficient separation and collection of photogenerated carriers. Moreover, doing‐induced bands within the bandgap of MoS_2_ enables an extrinsic broadband response to short‐wave infrared, paving the way for next‐generation multifunctional imaging systems and high‐performance photonic chips.

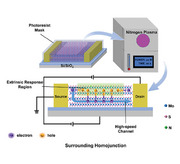

## 1 Introduction

In recent years, 2D materials, such as graphene,^[^ ^1^, ^2^, ^3^ ^]^ transition metal dichalcogenides,^[^ ^4^, ^5^, ^6^, ^7^, ^8^ ^]^ and black phosphorus,^[^ ^9^, ^10^, ^11^ ^]^ have garnered significant interest in the field of optoelectronics due to their unique structures, exceptional optoelectronic properties, and flexibility in regulation. Yunfeng Chen et al. utilized band‐engineered van der Waals heterostructures to construct unipolar barrier photodetectors capable of operating in both visible and mid‐infrared wavelengths. The unipolar barrier structure significantly suppresses the dark current in photodetectors by blocking the majority of carriers.^[^ ^12^ ^]^ Nikolaus Flöry et al. presented high‐performance vertical MoTe_2_‐graphene van der Waals heterostructure photodetectors integrated with planar silicon photonic waveguides, achieving record‐high bandwidth under moderate bias voltage.^[^ ^13^ ^]^ Nevertheless, the response range of these devices remains limited by the intrinsic bandgap of the 2D materials, significantly constraining their multi‐functional detection capabilities.

Several strategies have been explored to modify the bandgap of 2D material and broaden their spectral response range. Among these, ferroelectric‐gated photodetectors have been developed, utilizing ultrahigh electrostatic fields generated by polarized ferroelectric polymers to alter the energy bands, thereby extending their response wavelengths.^[^ ^14^, ^15^, ^16^, ^17^ ^]^ However, the photodetecting performance for these extended wavebands remains suboptimal and necessitates further enhancement. Additionally, tuning the bandgap by regulating the atomic ratio between molybdenum and sulfur in MoS_x_ has resulted in broadband detection spanning from 445 to 2717 nm.^[^ ^18^ ^]^ Despite this, the detectors suffer from relatively low carrier mobility and rich trapped states from the imperfections induced by the pulsed laser deposition technique. Moreover, extrinsic responses induced by vacancies have also been reported, as the vacancies introduce additional energy states within the bandgap and extend the responsive wavelength.^[^ ^19^, ^20^ ^]^ Despite these innovative approaches, the overall performance of these devices still requires substantial improvements. Achieving high‐performance extrinsic response in 2D materials poses a significant challenge, as a high concentration of extrinsic states and high carrier mobility are mutually exclusive and cannot be attained simultaneously.

In this work, we propose a novel configuration of the MoS_2_ device that features both a surrounding homojunction and a high‐speed underlying transport channel. The surrounding homojunction is achieved by localized nitrogen doping on the surface of n‐type MoS_2_, resulting in an isolated p‐type doped region semi‐encircled by pristine n‐type MoS_2_ material. This configuration creates p‐n junctions in both horizontal and vertical directions, enhancing the effective separation of photogenerated carriers. As a result, the device exhibits an exceptionally high photoresponsivity of 6.94 × 10^4^ A W^−1^ and a detectivity of 1.21 × 10^14^ Jones under 638 nm illumination, with a light on/off ratio of ≈10^7^ at a gate voltage of −27 V. Furthermore, Density Functional Theory (DFT) calculations corroborate that the introduction of nitrogen impurities forms extrinsic states within the bandgap of MoS_2_, extending the spectral response to the shortwave infrared (SWIR) band. With the aid of the surrounding homojunction structure and high‐speed underlying channel, the photogenerated carriers in the doping region are efficiently separated, transferred, and finally collected by the electrodes. Specifically, at the communication wavelength of 1550 nm, the device achieves a photoresponsivity of 34 A W^−1^ and a detectivity of 5.92 × 10^10^ Jones. The strategy of employing controllable doping and innovative structural design facilitates highly sensitive broadband detection for few‐layered 2D materials. The demonstrations of high‐contrast imaging in both the visible and SWIR bands, along with the integration with planar waveguides, signify their promising prospects for applications in post‐silicon and on‐chip integrated optoelectronics.

## 2 Results and Discussion

**Figure 1:**
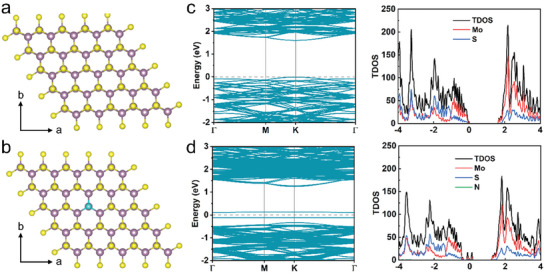
First‐principles calculations of pristine and N‐doped monolayer MoS_2_. a,b) Theoretical structure models of pure and N‐doped monolayer MoS_2_. c,d) Calculated electronic structures and DOS for pure and N‐doped monolayer MoS_2_.

We utilized the N_2_ plasma surface doping technique to alter the energy structure of MoS_2_. Ab initio DFT calculations were conducted to gain deeper insights into the effects of nitrogen incorporation into the lattice. **Figure** **1**a,b show the theoretical structure models of pure and N‐doped monolayer MoS_2_. In this structure, each molybdenum atom is precisely coordinated with six sulfur atoms to form a hexagonal plane. After N_2_ plasma doping, nitrogen atoms are introduced into the crystal lattice of MoS_2_ by replacing the sulfur atoms. We employed the plane wave basis to expand the wavefunctions,^[^ ^21^ ^]^ and the exchange‐correlation energy function was treated using the generalized‐gradient approximation as parametrized by Perdew–Burker–Ernzerhof.^[^ ^22^, ^23^, ^24^, ^25^ ^]^ Each self‐consistent electronic calculation was converged to within 10^−6^ eV, and the ionic and cell relaxation were iterated until the forces were less than 0.02 eV Å^−1^. Monkhorst‐Pack k‐mesh methods were used to produce k‐grids, and the k‐resolution was set to 0.02 × 2π. The plane‐wave basis of the kinetic cutoff energy set is 500 eV. The calculated band structure of pristine MoS_2_ is depicted in Figure 1c, considering the Brillouin zone along the high‐symmetry points of Γ–M–K–Γ. The conduction band minimum and valence band maximum align at the M point within the first Brillouin zone, indicating that monolayer MoS_2_ has a direct energy bandgap of 1.61 eV. This value agrees with previous calculation results,^[^ ^26^, ^27^ ^]^ and is also comparable with the experimental values.^[^ ^28^ ^]^ The Fermi level of pristine monolayer MoS_2_ is located at the valence band maximum. The density of states (DOS) in Figure 1c of pristine MoS_2_ was calculated within an energy range of −2 to 3 eV. The projected DOS (PDOS) of undoped MoS_2_ reveals that the *d*‐orbitals of Mo and *p*‐orbitals of S atoms mainly contribute to DOS for pure MoS_2_ material. Further details can be found in Figure S1 (Supporting Information), which provides a comprehensive account of the contribution of different orbitals of Mo and S atoms.

The band structure and DOS of N‐doped monolayer MoS_2_ are shown in Figure 1d. The incorporation of N atoms into the MoS_2_ material leads to significant changes in the band structure and DOS. The band structure of N‐doped monolayer MoS_2_ also shows a direct bandgap but with a slightly reduced bandgap. The calculated energy bandgap changes from 1.61 to 1.56 eV. Additionally, a downshift of both the valence band and conduction band is observed. There are two additional bands compared to the pure MoS_2_. It can be seen from the PDOS in Figure 1d that the two bands are contributed by Mo, S, and N atoms at the top of the valence band. The Fermi level lies between the two energy bands, and a shallow acceptor energy level above the valence band maximum indicates a p‐type doping effect. Figure S2 (Supporting Information) shows the detailed orbital composition of each band. Nitrogen doping in MoS_2_ alters both the electronic and optical properties of MoS_2_. The creation of impurity energy bands suggests an extended response wavelength into the shortwave infrared band.

The experimental results including the photoluminescence (PL) spectra in Figure S3 (Supporting Information) further indicate the changes in the composition, structure, and electronic properties of the materials caused by the doping process. The p‐type conduction behavior for nitrogen‐doped MoS_2_ has been reported in our previous work.^[^ ^29^ ^]^ Moreover, the energy dispersive spectroscopy (EDS) line profile in Figure S4 (Supporting Information), which shows the elemental distribution of S, Mo, and N along the coordinated arrow, reveals that nitrogen doping only occurs in the surface layers of MoS_2_, providing more opportunities for innovation in the device structure. X‐ray photoelectron spectroscopy (XPS) measurements are then conducted to further investigate the chemical valence states of the material. The normalized N 1*s*, Mo 3*d* and S 2*p*. Spectra shown in Figure S5 (Supporting Information), confirm the formation of Mo−N bonds in nitrogen‐doped MoS_2_ material.

**Figure 2:**
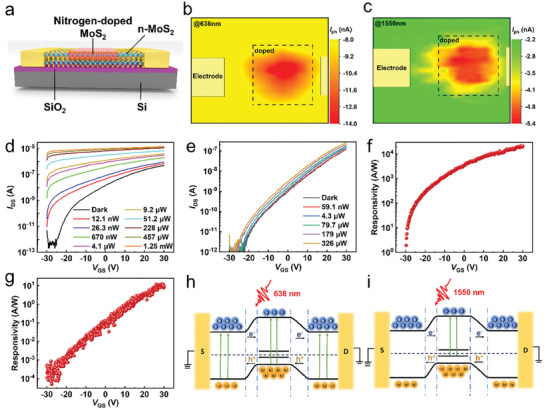
Gate‐sensitive photodetection of MoS_2_‐based device. a) Schematic illustration of the MoS_2_‐based device. b,c) Scanning photocurrent mapping of the MoS_2_‐based device under 638 and 1550 nm illumination with *V*_DS_ = −3 V and *V*_GS_ = 0 V, respectively. d) The *I*_DS_–*V*_GS_ characteristics at *V*_DS_  =  2 V under 638 nm laser. e) The *I*_DS_–*V*_GS_ characteristics at *V*_DS_  =  2.0  V under 1550 nm illumination. f) Gate dependence photoresponsivity at *V*_DS_ = 2 V under 638 nm laser. g) Gate dependence photoresponsivity at *V*_DS_ = 2.0 V under 1550 nm laser. h,i) Energy band diagrams of MoS_2_‐based device under 638 and 1550 nm irradiation at forward bias.

We then engineered a surrounding homojunction device by applying the selected area surface doping to the localized region of the naturally n‐type MoS_2_ channel. This configuration comprises a doped p‐type region semi‐enclosed by the pristine n‐type region, as shown in the schematic illustration of **Figure** **2**a. Its optical photograph and height curve are depicted in Figure S6 (Supporting Information). The localized p‐n homojunction create the boundaries between the nitrogen‐doped and undoped regions in both horizontal and vertical directions. As the carrier mobility for p‐type nitrogen‐doped MoS_2_ is about one order lower than the underlying n‐MoS_2_ channel,^[^ ^30^ ^]^ it is beneficial for the effective carrier separation and rapid transport of carriers through the high‐speed n‐type MoS_2_. The photocurrent mapping measurements were further used to elaborate the physical mechanism of the photodetection process. Figure 2b shows the scanned photocurrent mapping under 638 nm laser illumination. It demonstrates that a photogenerated current occurs across the entire material area when exposed to a 638 nm laser. This is attributed to the bandgap transition as the photon energy for the 638 nm laser surpasses the bandgap of MoS_2_. As a consequence of this energy transfer, electron‐hole pairs are generated due to the transition of electrons from the valence band to the conduction band. These pairs could be effectively separated by the applied electric field and then collected by the electrodes. However, the doped region exhibits a more pronounced photocurrent response. This suggests that the built‐in electric field created by p‐n junctions in both horizontal and vertical directions has a more significant effect on the separation and collection of the electron‐hole pairs, thereby forming a larger photocurrent signal at the doped region.

Figure 2c displays the photocurrent mapping under 1550 nm laser illumination, which differs from that of 638 nm laser. The photocurrent is observed only in the doped region, while no obvious photoelectric signal has been detected in the undoped area. The current in the undoped region at 1550 nm illumination is consistent with the dark current. This observation provides compelling evidence that the infrared band response stems from N_2_ plasma implantation. Doping nitrogen atoms onto the surface introduces impurity energy bands into MoS_2_ materials, resulting in an extrinsic response that extends the response range of MoS_2_ from the visible spectrum to the SWIR band. These findings are in good agreement with the computational results.

To modulate and improve the photoelectric performance of the device, the heavily doped p‐Si substrate was utilized as a back gate electrode. The gate voltage can precisely regulate the charge carrier density and adjust the electron occupancy of the underlying n‐type MoS_2_ channel, while the upper p‐type doping region is less affected due to the electrostatic screening.^[^ ^31^, ^32^ ^]^ The transfer curves obtained under 638 nm laser excitation with different powers are depicted in Figure 2d. Similar to previously reported MoS_2_ devices conducted with Pd or Au electrodes, the device exhibited typical n‐type behavior with a low cut‐off current of 10^−13^ A and a high saturation current of 10^−7^ A, resulting in an impressive modulation ratio of 10^6^. Despite being exposed to a low‐power 638 nm laser, the *I*_DS_ (drain current) still experienced a significant increase. The increase is particularly pronounced when the gate voltage is negative. Furthermore, as the laser power gradually increases, the *I*_DS_ continues to rise, indicating a dependence of photocurrent on the laser power. At the laser power of 1.25 mW, the device achieves an optical on/off ratio of 10^7^ at *V*_GS_ = −27 V. Figures S7 and S8 (Supporting Information) illustrate the time‐resolved photoresponse under 638 nm illumination at different optical powers and gate voltage, respectively. These phenomena suggest that the device is highly sensitive to both gate voltage and optical power. Figure S9 (Supporting Information) displays the transfer curves of an undoped MoS_2_ photodetector with different powers. In comparison, the performance of the doped device is significantly superior.

The transfer curve obtained under the 1550 nm laser, as depicted in Figure 2e, exhibits similar n‐type conductive behavior to that under the 638 nm laser. *I*_ds_ also increase with the increase in laser power. However, there is a significant difference in their transfer characteristics under different laser conditions. The transfer curve obtained under the 1550 nm laser shows a similar light on/off ratio at various gate voltages, while the light on/off ratio under 638 nm gradually decreases with the increase of voltage under. Besides, the photocurrent is nearly linearly dependent on the laser power. The primary reason is that the infrared waveband response only originates from the doping region on the surface of MoS_2_, which is beyond the tunability range of the back gate voltage. Only the photogenerated electrons that are transferred to the underlying n‐type MoS_2_ driven by the built‐in electric field of the surrounding homojunction are electrostatically tuned by the gate voltage. Therefore, the photoresponsive region to the infrared waveband is distinct from the gate‐voltage tuned transport channel, resulting in a similar current profile for the transfer curves under different laser power conditions, just like the one measured in the dark.

In order to further elaborate the impact of gate voltage on the photoelectric performance, the plots illustrating the relationship between photoresponsivity and gate voltage are plotted in Figure 2f,g. It is found that the photoresponsivity of the device is notably influenced by the gate voltage, both for visible light and infrared waveband detection. Considering that an increase in gate voltage leads to higher responsivity, it is reasonable to infer that the increased electron density induced by the gate voltage in the underlying n‐type MoS_2_ has enlarged the built‐in field, thereby enhancing the efficiency of carrier separation. The maximum responsivity, reaching ≈2.1 × 10^4^ A W^−1^ for 638 nm laser detection, has been achieved at *V*_GS_ = +30 V. These findings highlight the crucial role of gate voltage in the photoresponsivity of the device. Remarkably, the responsivity in logarithmic coordinates for 1550 nm exhibits an approximately linear relationship with the gate voltage, in contrast to the responsivity for 638 nm. The maximum responsivity of ≈9 A W^−1^ is also achieved at *V*_GS_ = +30 V. It is worth mentioning that the data points plotted in Figure 2g appeared to be more discrete compared to those in Figure 2f. This discreteness can be attributed to the low signal‐to‐noise ratio of the device when operating in the SWIR range. The gate‐voltage‐dependent response clearly indicates that a higher forward gate voltage is necessary to achieve a larger photodetector response.

The photodetection mechanism is further elucidated in Figure 2h,i. In the surrounding homojunction MoS_2_ device, the nitrogen‐doped p‐type region is partially enclosed by pristine n‐type MoS_2_, forming a photoconductive structure. The built‐in electric field induced by the homojunction accelerates the separation of photogenerated carriers. Meanwhile, a high‐speed underlying channel is still retained, enabling rapid carrier transport. When the device is irradiated with a 638 nm laser, as shown in Figure 2h, numerous electron‐hole pairs are generated. Some carriers are collected by the electrodes across the n‐p‐n junction in the upper channel due to the large external forward bias, while more carriers are collected via the high‐speed underlying channel of MoS_2_. When exposed to a 1550 nm laser, an extrinsic transition occurs from the impurity band to the conduction band of MoS_2_, as depicted in Figure 2i. Consequently, the photogenerated carriers are excited only in the doped region. For the upper n‐p‐n channel, only noise electrons can pass through when a large external forward bias is applied,^[^ ^33^ ^]^ while both photogenerated electrons and holes can be collected by the electrodes. However, due to the reduced mobility in the nitrogen‐doped p‐type region, the efficiency of carrier collection through the upper n‐p‐n channel is significantly low. Due to the high‐speed underlying n‐MoS_2_ channel, the photogenerated electrons separated by the vertical built‐in electric field can be efficiently collected.

**Figure 3:**
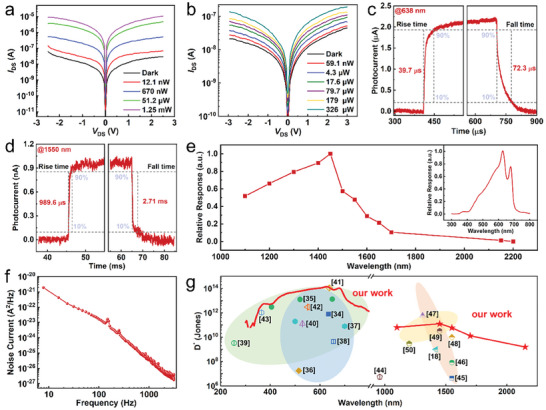
High sensitivity and broadband photoresponse of the device. a,b) *I*_DS_–*V*_DS_ characteristics at *V*_GS_ = 25 V under 638 and 1550 nm lasers, respectively. c,d) Time‐resolved photoresponse at 638 and 1550 nm laser, respectively. The *τ*_rising_ and *τ*_falling_ for 638 nm are 39.7 and 72.3 µs at *V*_DS_ = −2 V and *V*_GS_ = −20 V, and for 1550 nm are 0.99 and 2.71 ms at *V*_DS_ = −1 V and *V*_GS_ = 0 V. e) Extracted wavelength‐dependent photoresponsivity of the MoS_2_‐based photodetector under shortwave infrared. Inset: wavelength‐dependent photoresponsivity under visible light. Measurements were conducted at *V*_DS_ = −5 V and *V*_GS_ = −15 V in ambient air. f) Current noise power spectrum measured from 1 Hz to 3 kHz. g) Room‐temperature specific detectivity *D* ^*^ as a function of wavelength for various MoS_2_‐based devices and conventional shortwave infrared materials.

The power‐dependent output curves were further measured at *V*_GS_ = +25 V. **Figure** **3**a illustrates the output curves under 638 nm laser illumination, providing a comparison between output characteristics under dark conditions and various power intensities of laser illumination. The dark current is ≈3 × 10^−8^ A when an external voltage of ±2.5 V is applied. The curves are nearly symmetrical with respect to the y‐axis due to the inherent design of the photoconductive structure. The device is responsive to 638 nm light across a wide range of power levels spanning at least five orders of magnitude, from nanowatts to milliwatts. The current under the irradiation of a 12.1 nW laser is clearly distinct from the dark current, indicating the exceptional sensitivity of this surrounding homojunction device. The calculated optimal responsivity is 6.94 × 10^4^ A W^−1^ at *V*_GS_  =  25 V and *V*_DS_  =  2.5 V. The extracted power‐dependence photoresponsivity is displayed in Figure S10 (Supporting Information). The power‐dependent output curve of the undoped device is shown in Figure S11 (Supporting Information). Similar to the transfer curve, the variation in photocurrent with optical power is smaller for the undoped device compared to the doped device.

The *I‐*‐*V* characteristics under the 1550 nm laser are carefully studied by adjusting the laser power from 59 nW to 326 µW. The results, presented in Figure 3b, show that the output curves resemble those obtained under 638 nm illumination, displaying fine symmetry between the forward and reverse currents. The photocurrent exhibits a consistent trend with varying optical powers of 1550 nm laser, increasing in tandem with both the external bias voltage and the optical power. This indicates that the device maintains high sensitivity and responsiveness across a broad range of light intensities, originating from the extrinsic response. The strong dependency relationship between the photocurrent and both the external bias and the optical power demonstrates efficient photocarrier generation and collection from the high‐speed underlying n‐MoS_2_ channel. In particular, the obtained responsivity is as high as 34 A W^−1^, almost three orders of magnitude higher than that of the device fabricated through regulation of the atomic ratio.^[^ ^18^ ^]^ This is thanks to our facile design of a high‐speed channel. The extracted photoresponsivity as a function of power is shown in Figure S12 (Supporting Information).

Response time is also a critical parameter used to evaluate the response speed of a detector. It typically refers to the rise time (***τ*_rising_**) and decay time (***τ*_falling_**), defined as the duration taken for the photocurrent to increase from 10% to 90% and decrease from 90% to 10% after the laser is turned on or off. Under negative gate and bias, the response time under 638 nm laser illumination has been measured and plotted in Figure 3c. The rise time is 39.7 µs and the decay time is 72.3 µs. This is attributed to the special surrounding homojunction structure of our devices, which promotes the fast separation of photogenerated carriers. Additionally, the decay time is longer than the rise time due to the presence of the photothermal effect. Response time at *V*_GS_  =  20 V and *V*_DS_  =  2.0 V is represented in Figure S13 (Supporting Information).

The response time in the shortwave infrared, as illustrated in Figure 3d, is notably different from that observed in visible light. Upon illumination, the drain current displays a gradual rise, taking ≈0.98 ms to reach its maximum. The decay time was observed to be ≈2.71 ms. The response speed is about one order slower compared to 638 nm light detection. The prolonged response time can be attributed to several factors, including the presence of an isolated extrinsic responsive region for the infrared waveband, situated away from the electrodes. Additionally, the weakened mobility, influenced by lattice distortions and defects resulting from doping, likely contributes to the slower response.

The extrinsic and surrounding homojunction device demonstrates remarkable photoresponsivity in the visible region, as well as an extended response waveband to shortwave infrared (450–2200 nm). The response spectrum presented in Figure 3e shows the relative photoresponsivity for the wavelength ranging from 1100 to 2200 nm. The data reveal a significant increase in responsivity as the illumination wavelength shifts from 1100 to 1450 nm. Beyond 1450 nm, the responsivity declines more rapidly and shows minimal fluctuations in the range from 1450 to 1700 nm. Consequently, a peak photoresponse is observed ≈1450 nm. This response in the shortwave infrared band could be attributed to the extrinsic transition from the impurity band to the conduction band. The inset in Figure 3e shows the relative photoresponse spectrum for visible light within the wavelength range of 300–800 nm. The spectrum features two prominent peaks centered at 625 and 675 nm, originating from the direct transition from the valence band to the split K points (K and K') in the Brillouin zone. Subsequently, the responsivity decreases sharply ≈700 nm, as it surpasses the bandgap absorption of the MoS_2_ materials. The relative responsivity spectra with different bias and gate voltage in the visible region are shown in Figure S14 (Supporting Information).

To assess the sensitivity of our MoS_2_ photodetectors, we first measured the current−noise density spectra at *V*_DS_ = 2.5 V and *V*_GS_ = 25 V, as displayed in Figure 3f. Typically, the total noise current (*i*_n_) of the detector includes 1/*f* noise, shot noise (*i*_s_), and thermal noise (*i*_t_). At low frequencies, the dominant contributor to the noise current is 1/*f* noise, which arises from the fluctuations of carriers being trapped and detrapped by defects and disorders, commonly found in 2D materials. The shot noise is determined by the expression *i*_s_ = (2*qI*_d_Δ*f*)^1/2^, where *I*_d_ is the dark current and Δ*f* is the bandwidth. With a dark current of ≈3 × 10^−8^ A at *V*_DS_ = 2.5 V and *V*_GS_ = 25 V, the shot noise is calculated to be 98.0 fA Hz^−1/2^ for a bandwidth of 1 Hz. Meanwhile, the thermal noise is estimated to be 14.1 fA Hz^−1/2^ based on the expressions *i*_t_ = (4*k*_B_ *T*Δ*f*/*R*_Ω_)^1/2^, where *k*_B_ is the Boltzmann constant, *T* the thermodynamic temperature, and *R*_Ω_ the resistance.

We then calculated the noise equivalent power (NEP), another crucial figure of merit for assessing photodetector sensitivity. NEP is defined as the ratio of the measured noise current (*i*_n_) to the corresponding responsivity. In general, a lower NEP indicates a higher capability to distinguish between the desired signal and unwanted noise. For our MoS_2_ photodetectors, the optimal NEP values are calculated to be 9.69 × 10^−19^ W Hz^−1/2^ at 638 nm and 1.98 × 10^−15^ W Hz^−1/2^ at 1550 nm, respectively. These results indicate that the photodetectors possess exceptional sensitivity and can detect extremely low power levels at these specific wavelengths.

The specific detectivity (*D* ^*^) is calculated using the formula *D* ^*^ = (*A* · Δ*f*)^1/2^ *R*/*i*_n_, where *A* represents the active area of the device. The wavelength‐dependent specific detectivities *D* ^*^ of our MoS_2_ device and other 2D materials‐based detectors are plotted in Figure 3g.^[^ ^34^, ^35^, ^36^, ^37^, ^38^, ^39^, ^40^, ^41^, ^42^, ^43^, ^44^, ^45^, ^46^, ^47^, ^48^, ^49^, ^50^ ^]^ The specific detectivity of our device is as high as 1.21 × 10^14^ Jones at 638 nm and 5.92 × 10^10^ Jones at 1550 nm, which are comparable to state‐of‐the‐art commercial photodetectors. Furthermore, Tables S1 and S2 (Supporting Information) summarize all the relevant optoelectronic parameters, providing a comprehensive overview of the optoelectronic properties in visible light and SWIR light. MoS_2_ samples have a degree of stability in ambient air. Several doped MoS_2_ devices were sealed in a vacuum bag and stored in a drybox filled with air. Almost a year later, they still demonstrated photoresponse under visible illumination, with almost no change in their photoresponse over the subsequent 10 days (see Figure S15, Supporting Information). Due to their exceptional optoelectronic performance and compatibility with existing technologies, our extrinsic and surrounding homojunction MoS_2_ photodetectors have great potential for imaging applications in both the visible and shortwave infrared bands.

**Figure 4:**
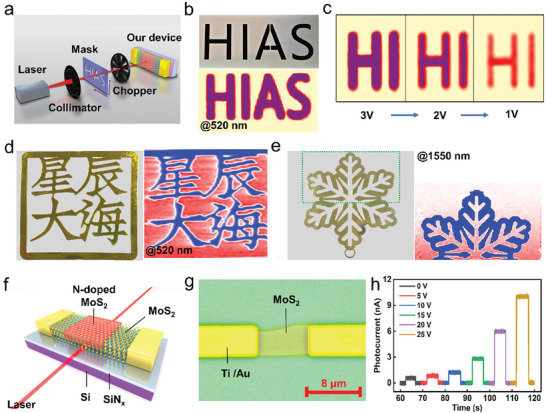
Photoelectric imaging applications and integration with waveguides of the device. a) A schematic diagram of the imaging system, where the devices are used as imaging pixels. b) The mask of object “HIAS” (top picture) and the corresponding high‐resolution imaging (bottom picture) at 520 nm. c) The imaging of object “HI” at different *V*_DS_ of 3, 2, and 1 V at 520 nm. d) The object “星辰大海” (left picture) and the corresponding higher‐resolution imaging (right image) at 520 nm. e) The object “snowflakes” (left picture) and its imaging (right image) at 1550 nm. f) A schematic diagram of the integration of the detector with waveguides. g) Optical photograph of the waveguide integrated device. h) The on/off photoresponse of the waveguide integrated device at different gate voltages.

Optical imaging measurements were conducted to evaluate the performance of the photodetectors for various imaging applications. **Figure** **4**a illustrates an imaging system that includes a laser, collimator, mask, chopper, and the photodetectors. The mask in this system can move continuously in both the horizontal and vertical directions, denoted as the X and Y directions, respectively. The movement is achieved through a high‐precision stepping motor, covering the entire object area point‐by‐point. A focused, detectable laser beam illuminates the object, and with the assistance of a preamplifier and a lock‐in amplifier, the photocurrent signal from the detector is recorded by a computer along with the position coordinates. Throughout the imaging measurement process, the optical signal is converted into an electrical signal by the detector.

To assess the imaging capability of the device, a hollow “HIAS” metal plate (24 mm × 10 mm) was selected as the imaging object. The 520 nm laser transmits through the hollowed‐out portion of the metal plate to the detector, forming an image of the character “HIAS”, as shown in Figure 4b. The image exhibits remarkable clarity and precision, with a resolution better than 0.5 mm, determined by the step distance.

By altering the external bias, the response of the detector could be tuned for 520 nm illumination. This variation in response results in noticeable differences in the clarity of the imaging, as depicted in Figure 4c. The external bias could help to improve the imaging quality for different scenarios.

After conducting optical imaging of simple objects, we further proceeded with imaging a more complex object. Figure 4d shows an object with Chinese characters and its imaging. The details were accurately captured, confirming the successful imaging of the complex object. This high performance foresees the potential of our device for applications in high‐resolution imaging, optical sensing, and advanced microscopy.

Infrared imaging technology has numerous advantages over visible light imaging, such as non‐contact detection, high spatial resolution, high sensitivity, real‐time capability, and wide applicability. The high contrast image (Figure 4e) of the “snowflower”‐like pattern was obtained using the detector with a 1550 nm laser. This image shows the intricate details of the snowflower's tiny branches, demonstrating that our extrinsic detection strategy through the surrounding homojunction device may contribute to the advancement of next‐generation infrared imaging.

Moreover, with the development of optical networks, integrated optoelectronic chips have shown wide application prospects in areas such as optical communications,^[^ ^51^, ^52^ ^]^ optical interconnections,^[^ ^53^, ^54^ ^]^ real‐time sensing,^[^ ^55^, ^56^ ^]^ integrated spectrometers,^[^ ^57^ ^]^ and optogenetics.^[^ ^58^ ^]^ An essential component of the optoelectronic chip is the waveguide‐integrated photodetector, which serves as the joint between photonic and electronic chips, converting the optical signals into electrical signals within the waveguide. 2D material detectors offer great advantages for waveguide integrated photodetectors due to their atomic‐scale thickness, lack of dangling bonds, and ease of integration. Encouraged by the device's response at 1550 nm, we integrated the MoS_2_ device onto a silicon nitride planar waveguide. Figure 4f shows the schematic structure of the integrated device. The 1550 nm laser is incident on the side cross‐section of the silicon nitride waveguide via an optical fiber and is detected once it passes through the channel of the MoS_2_ device. Figure 4g presents an optical photograph of the waveguide integration device, with a scale bar of 8 µm. The gate‐dependent photocurrent, as depicted in Figure 4h, demonstrates that the waveguide response of the device is successfully achieved and can be controlled by the gate voltage. The excellent photoelectric performance of the waveguide integrated structure makes it a promising candidate for application in next‐generation photonic chips. In future work, we aim to integrate our device with a line waveguide to realize a complete waveguide chip. Additionally, the integrated waveguide structure is expected to be more efficient due to the improved signal transmission and reduced signal degradation.

## 3 Conclusion

In summary, a high‐quality surrounding homojunction MoS_2_ device was successfully fabricated via N_2_ plasma implantation. The device exhibits a remarkable photoresponsivity of 6.94 × 10^4^ A W^−1^ and a specific detectivity of 1.21 × 10^14^ Jones under 638 nm illumination, with the optical on/off ratio of the device up to seven orders of magnitude at *V*_GS_ = −27 V. Notably, the response wavelength of MoS_2_ materials could be extended from visible light to SWIR through the extrinsic response. Consequently, the MoS_2_ device achieves a photoresponsivity of 34 A W^−1^ and a specific detectivity of 5.92 × 10^10^ Jones under the 1550 nm illumination. Our MoS_2_ photodetector was further applied for imaging in both visible and SWIR bands, and integrated with waveguides. These findings not only pave the way for the challenging extrinsic detection in low‐dimensional materials but also provide new avenues for the development of high‐performance photonic chips and multifunctional imaging devices.

## 4 Experimental Section

### The Pristine n‐type MoS_2_ FET Device

The few‐layered MoS_2_ nanoplates used in this study were mechanically exfoliated from bulk MoS_2_ materials via a scotch‐tape exfoliation method. These target MoS_2_ nanoplates were then found and positioned by an optical microscope. A precise fixed‐point transfer technique was employed to transfer these target nanoplates onto highly p‐doped Si/SiO_2_ substrates and SiN_x_ waveguides respectively, using polydimethylsiloxane. These substrates with MoS_2_ nanoplates were soaked in hot acetone for 2 h and cold acetone for 30 min to eliminate any residual adhesive. Subsequently, the standard laser direct writing (LDW) process was carried out to fabricate drain, source, and gate electrode patterns. A high vacuum evaporation system was utilized to deposit Cr/Au (10/70 nm) electrodes with a rate of 0.5 Å s^−1^ and under the vacuum of 10^−5^ Pa. The pristine n‐type MoS_2_ FET Devices were successfully fabricated.

### N_2_ Plasma Surface Doping

A standard LDW process was used to form mask patterns on localized areas of the surface of the n‐type MoS_2_ FET devices. The treated devices were subjected to N_2_ plasma surface doping in a general plasma cleaner (Pluto M, Shanghai Peiyuan Instrument Equipment Co., Ltd.). During this process, the samples were placed in a vacuum chamber, operating at 50 W for 30 s. The flow rate of N_2_ gas was consistently maintained at 200  mL min^−1^. Afterward, the doped devices were annealed in a dual‐temperature tube furnace at 300 K for 2 h.

### Material Characterization

The optical microscopy images for the MoS_2_‐based devices on Si/SiO_2_ substrates and SiN_x_ waveguides were captured by an OLYMPUS microscope. EDS analysis was conducted with a field emission gun Tecnai F20 microscope operating at 200 kV. PL spectra were obtained using the fluorescence module within the Rapid Imaging Raman Microscope device. The thickness of N‐doped MoS_2_ material was characterized by an atomic force microscope (Dimension ICON). The chemical valence states and the stoichiometry of N‐doped MoS_2_ material were investigated using XPS (Thermal, ESCALAB250Xi).

### Electrical and Photoresponsivity Characterizations

Before electrical and photoresponsivity characterizations, the electrode micro pads were linked to the PCB module using a wedge wire bonder (West Bond, model 7476D). A back gate technology was utilized to apply gate voltage. Subsequently, both electrical and optoelectronic properties were characterized in ambient air at room temperature. A highly sensitive dual‐channel digital source meter (Keithley 6482) applied the bias and measured the photocurrent simultaneously, while another digital source meter (Keithley 2602) was used for applying the gate voltage. During the measurement process, the devices were affixed to a sample holder to enhance stability and precision. The effective light power density (*P*_eff_) was calculated by the formula *P*_eff_ = *P*_in_ × *A*_device_/*A*_spot_, where *A*_device_ = 3 µm × 3.5 µm = 10.5 µm^2^, and the laser spot diameter measured 135 µm. It is evident that the spot area substantially exceeds the device area, ensuring minimal disruption to the device's symmetry. The photocurrent mapping was conducted by scanning the pressure point console both horizontally and vertically while the devices were irradiated with a focused modulated laser. The resulting photocurrent signal was obtained using a lock‐in amplifier (MStarter 200). Relative responsivity in the visible region was measured using a grating spectrometer at Zhejiang University, while for the SWIR region, it was extracted from data measured with a continuous femtosecond laser. The noise spectral density was measured using an FFT Spectrum Analyzer (SR770) in a dark and metal‐shielded environment.

## Conflict of Interest

The authors declare no conflict of interest.

## Supporting information



Supporting Information

## Data Availability

The data that support the findings of this study are available from the corresponding author upon reasonable request.
