# Supplementary material for: An Ultrasensitive and Broad‐Spectrum MoS2 Photodetector with Extrinsic Response Using Surrounding Homojunction
Source: Adv Sci (Weinh). 2024 Oct 16;11(45):2408299. doi: 10.1002/advs.202408299 (PMC11615792; doi:10.1002/advs.202408299)
Supplement: Supplementary file 1 — Supporting Information [file ADVS-11-2408299-s001.docx]

Supporting Information

An ultrasensitive and broad-spectrum MoS_2_ photodetector with extrinsic response using surrounding homojunction

Xiaoyan Liu^#^, Jiaqi Zhu^#^, Yufeng Shan*, Changlong Liu, Changyi Pan, Tianning Zhang, Chixian Liu, Tianye Chen, Jingwei Ling, Junli Duan, Feng Qiu, Saqib Rahman, Huiyong Deng* and Ning Dai*

**Main contents**

1. **The contribution of different orbitals of Mo and S atoms to density of states in pristine few-layer MoS_2_ materials.**
2. **The contribution of different orbitals of Mo, S and N atoms to density of states in doped few-layered MoS_2_ materials.**
3. **Photoluminescence (PL) spectrum of pristine and doped MoS_2_ material.**
4. **The EDS line profile of doped MoS_2_ materials.**
5. **The normalized XPS spectra of doped MoS_2_ material.**
6. **The optical photograph and height curve of our device.**
7. **Optical switching under a 638 nm laser with different optical powers.**
8. **Optical switching with different gate voltage under a 638 nm laser.**
9. **The transfer curves of undoped MoS_2_ device.**
10. **Extracted power dependence photoresponsivity in the visible light.**
11. **The power-dependent output curves of the undoped device.**
12. **Extracted power dependence photoresponsivity in the shortwave region.**
13. **Response time under 638 nm laser illumination at *V*_GS_  =  20 V and *V*_DS_  =  2.0 V.**
14. **Relative response in the visible region.**
15. **Stability of a doped MoS_2_ photodetector.**
16. **Comparison of the relevant optoelectronic parameters for different 2D MoS_2_-based photodetectors on the visible light.**
17. **Comparison of the relevant optoelectronic parameters for different 2D MoS_2_-based photodetectors and photodetectors based on other materials for SWIR region.**
18. **The contribution of different orbitals of Mo and S atoms to density of states in pristine few-layer MoS_2_ materials.**

In pristine few-layered MoS_2_ materials, both S and Mo atoms contribute to the density of states. However, the contribution varies across different orbitals of each atom. Among S atoms, the *p* orbitals, particularly *p*_z_ and *p*_x_ orbitals, exert a notable impact, while *p*_y_ orbitals have minimal effect. For Mo atoms, the *d* orbitals play a more significant role, particularly *d*_z2_, *d*_xz_, and *d*_x2_-_y2_ orbitals, while *d*_xy_ and *d*_yz_ orbitals have an almost negligible effect.


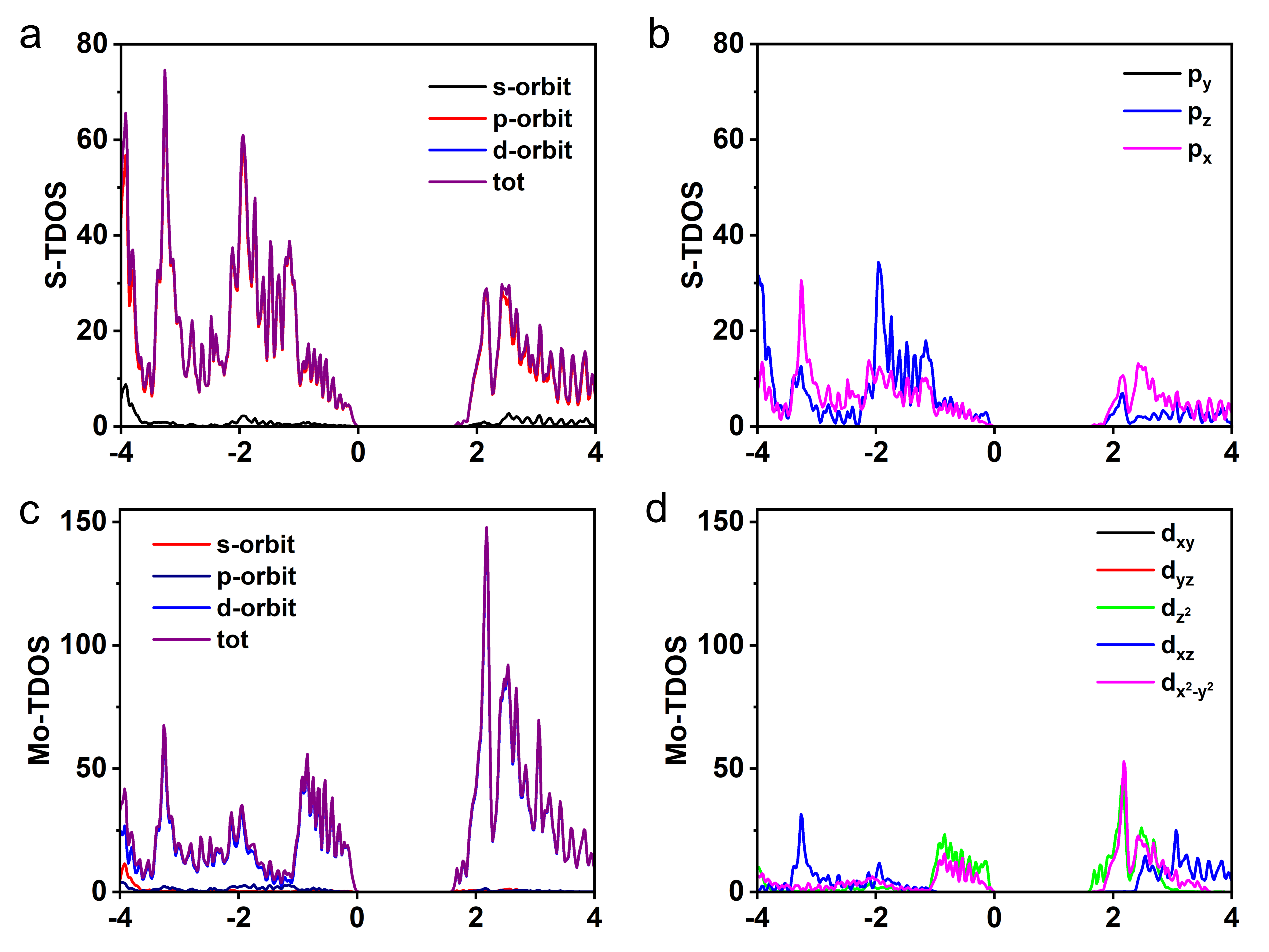


1. **Figure S1.** The contribution of different orbitals of Mo and S atoms in pristine few-layered MoS_2_ materials. (a, c) The contribution of different orbitals of S and Mo atoms, respectively. (b, d) The contribution of different *p* orbitals for S atoms and different *d* orbitals for Mo atoms. **The contribution of different orbitals of Mo, S and N atoms to density of states in doped few-layered MoS_2_ materials.**

**
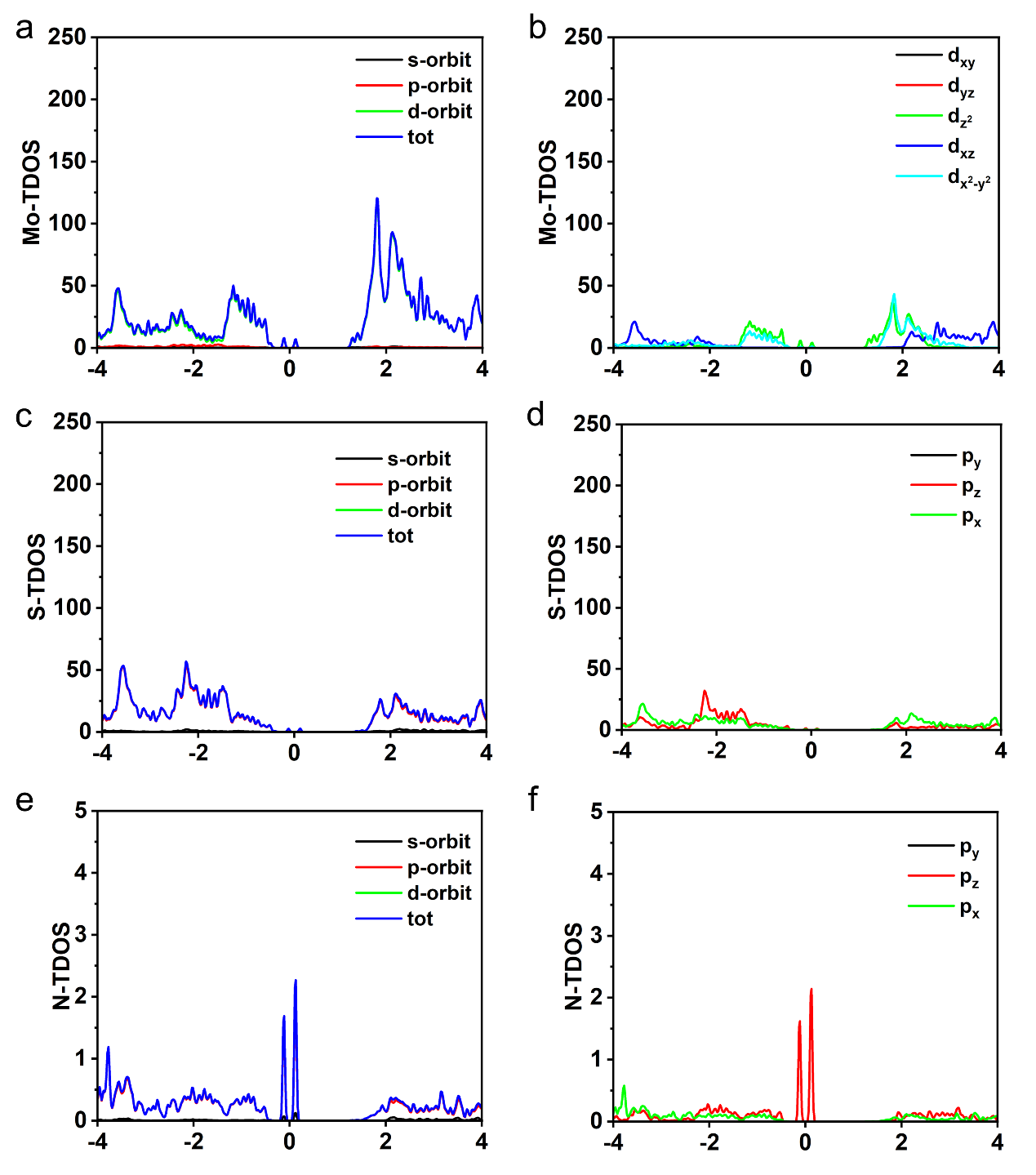
**

1. **Figure S2.** The contribution of different orbitals of Mo, S and N atoms in doped few-layered MoS2 materials. The contribution of different orbitals of (a) Mo atoms, (c) S atoms, and (e) N atoms. The contribution of different (b) d orbitals of Mo atoms, (d) p orbitals of S atoms, and (f) p orbitals of N atoms.**Photoluminescence (PL) spectrum of pristine and doped MoS_2_ material.**

The photoluminescence (PL) spectrum of MoS_2_ material undergoes significant alteration due to the N_2_ plasma implantation process. Prior to the implantation, the pristine MoS_2_ material displays two distinct peaks labeled peak A and peak B. They are centered at 677nm (1.83 eV) and 632nm (1.96 eV) respectively, with a separation of approximately 130 meV. These peaks correspond to direct transitions from valence band to the split K points (K and K') in the Brillouin zone. For doped MoS_2_ material, peak A is centered at 662nm (1.87 eV), presenting a distinct blue shift. This is attributed to the recombination of excitons without forming trions after nitrogen doping.^[1-2]^ The finding illustrates the changes in composition and electronic properties of the materials caused by the doping process, highlighting the potential of N_2_ plasma implantation to regulate the optical properties of MoS_2_ materials and indicating significant prospects for the development of next-generation optoelectronic devices.


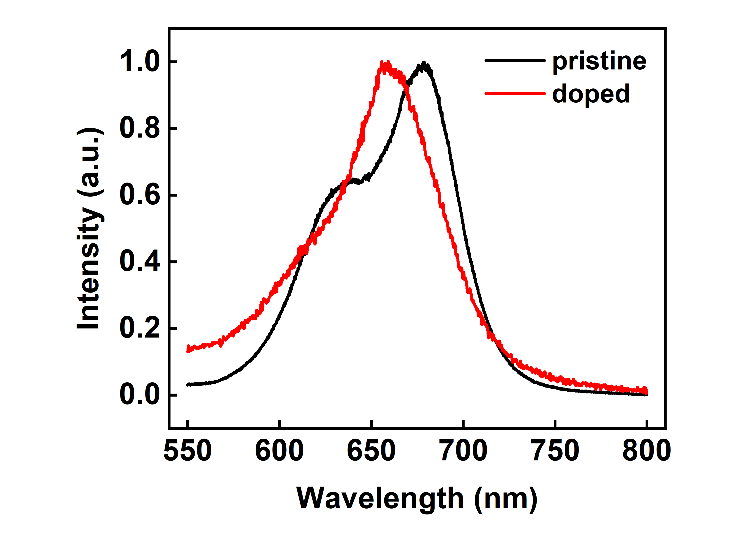


**Figure S3.** The PL spectrum of MoS_2_ material before and after N plasma doping.

1. **The EDS line profile of doped MoS_2_ materials.**

the energy dispersive spectroscopy (EDS) line profile shows the elemental distribution of S, Mo, N along the coordinated arrow. Specifically, at the interface between Au and MoS_2_, there is a notable decrease in the S Kα line, while a gradual increase in the N Kα line is observed. The Mo signal at the interface maintains relative stability. These findings suggest that the doping of N impurities is accompanied by the creation of S vacancies. Additionally, N elements are predominantly found on the upper surface of MoS_2_, beneath the gold layer. Several comparable studies have reported that the underlying layer is probable to be nitrogen-free for thicker MoS_2_ samples with more than six layers.^[3]^


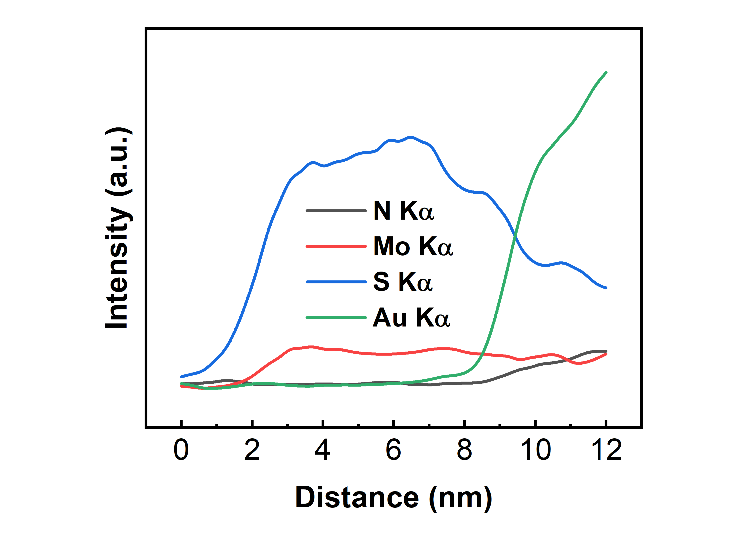


**Figure S4.** The EDS line profile of doped MoS_2_ materials.

1. **The normalized XPS spectra of** **doped MoS_2_ material.**

XPS measurements were performed to further quantify the relative concentrations of various elements and investigate the elemental valence states in the doped MoS_2_ material. The atomic percentage of nitrogen in the material is approximately 0.66%, indicating the successful introduction of nitrogen into the MoS_2_ material. Furthermore, the normalized N 1*s* spectrum is also provided (Figure S5a). The spectrum displays partial overlap with the Mo 3*p*_3/2_ peaks, centered around 395.5 eV. The other peak centered at around 397.5 eV corresponds to the N−Mo bonds.^[4,5]^ These XPS results confirm the incorporation of nitrogen into the MoS_2_ lattice and the formation of N-doped MoS_2_ nanosheets. Moreover, the high-resolution XPS spectra of Mo 3*d* is presented to recognize the bonding states of Mo (Figure S5b). Peaks centered at 229.6 eV (3*d*_5/2_) and 232.8 eV (3*d*_3/2_) are typical Mo-S bonding of MoS_2_.^[6,7]^ The other two peaks centered at 233.1 eV (3*d*_5/2_) and 236.1 eV (3*d*_3/2_) are associated with Mo^6+^ in Molybdenum oxides.^[8]^ In XPS results of S 2*p* (Figure S5c), there are two peaks centered at 162.4 eV (2*p*_3/2_) and 163.6 eV (2*p*_1/2_) respectively, and no characteristic peaks related to the S-N bonding.


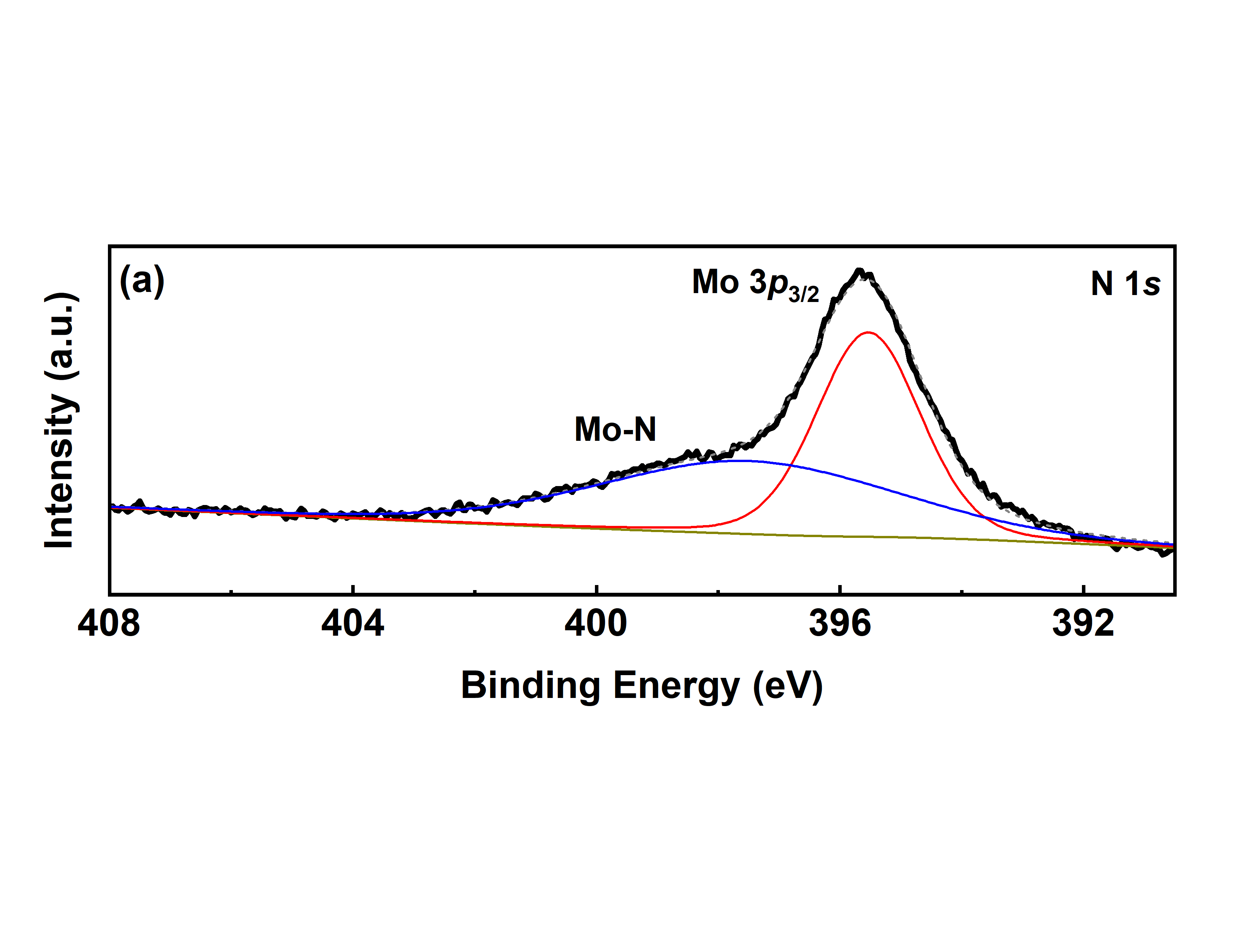

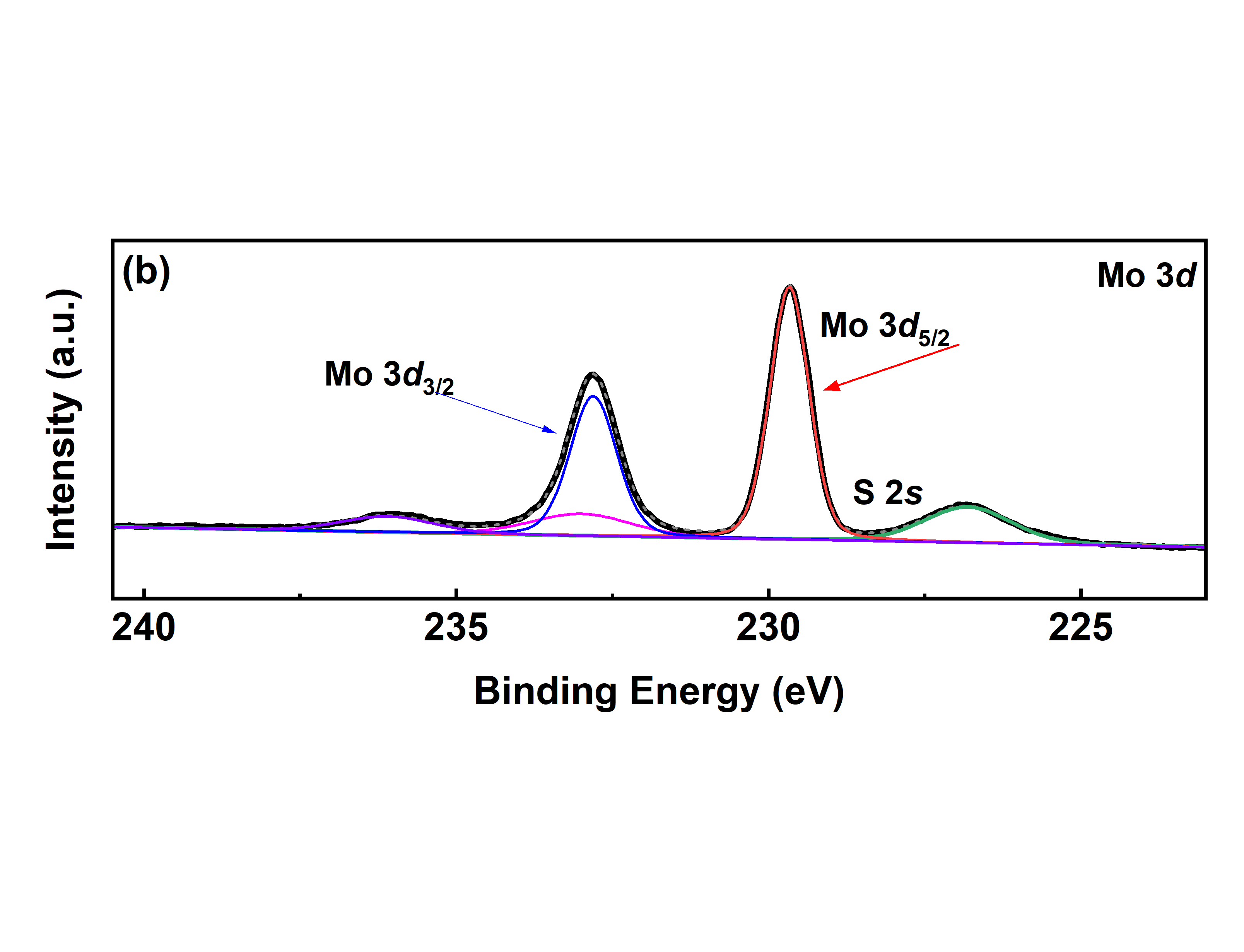

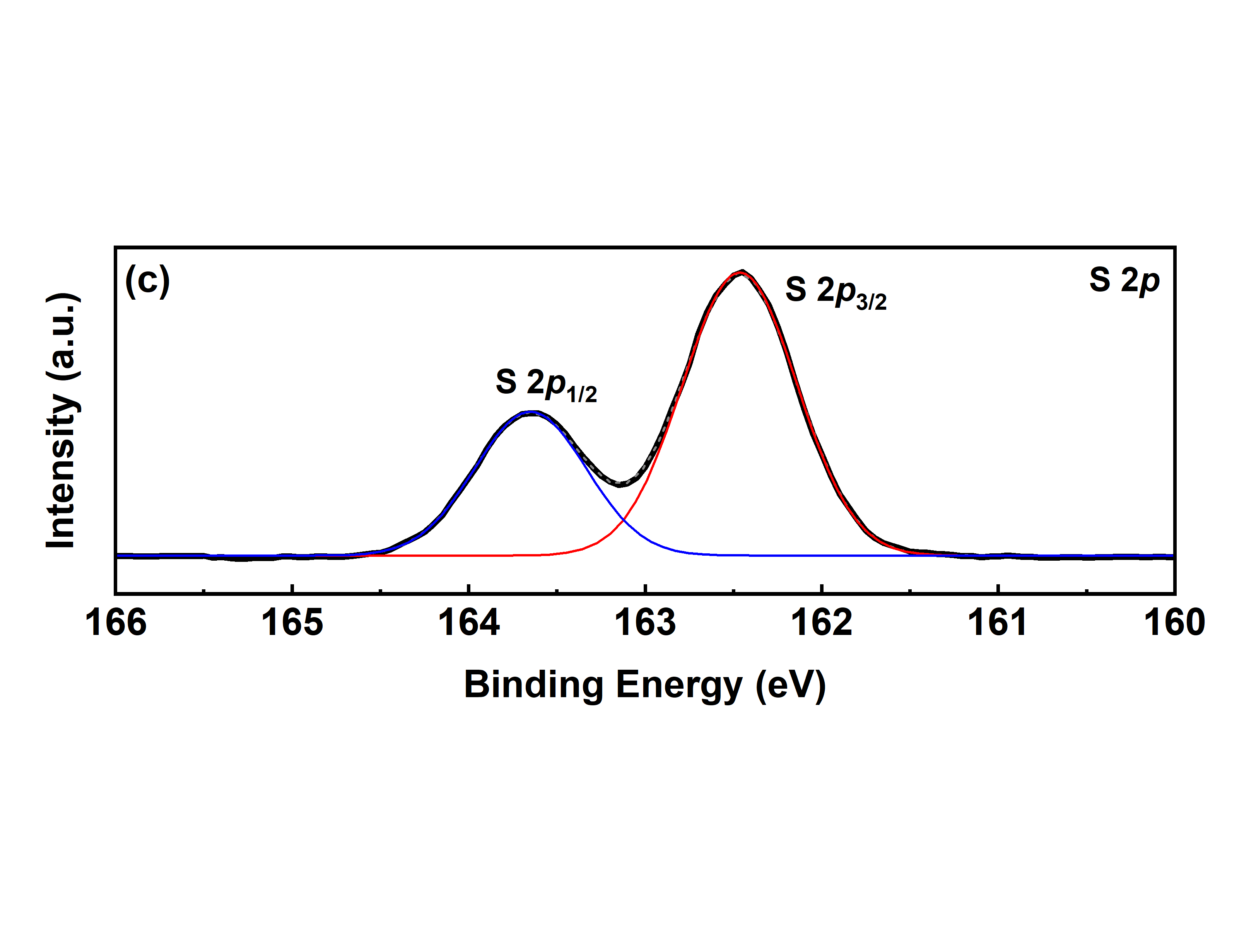


**Figure S5.** The normalized XPS spectra of doped MoS_2_ material. (a) N 1*s*, (b) Mo 3*d*, (c)S 2*p*.

1. **The optical photograph and height curve of our device.**

Figure S6a shows the optical photograph of the MoS_2_ device, with a scale bar of 5 μm. The height profile across a MoS_2_ step is displayed in Figure S6b, indicating that the thickness of the used MoS_2_ material is approximately 10.7 nm, corresponding to about 16-monolayer.

**
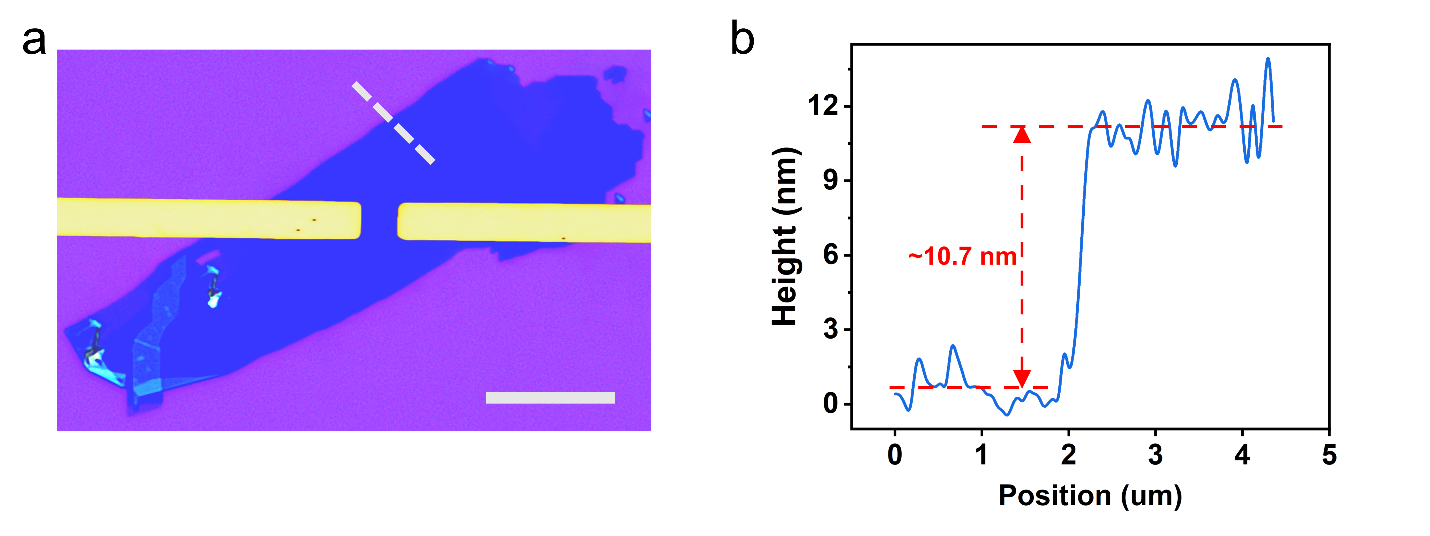
**

**Figure S6.** The optical photograph and height curve. (a)The optical photograph of our device, scale bar 10 μm. (b) height curve (see the white dashed line across a MoS_2_ step in the figure S6a) indicating the thickness of ~10.7 nm.

1. **Optical switching under a 638 nm laser with different optical powers.**


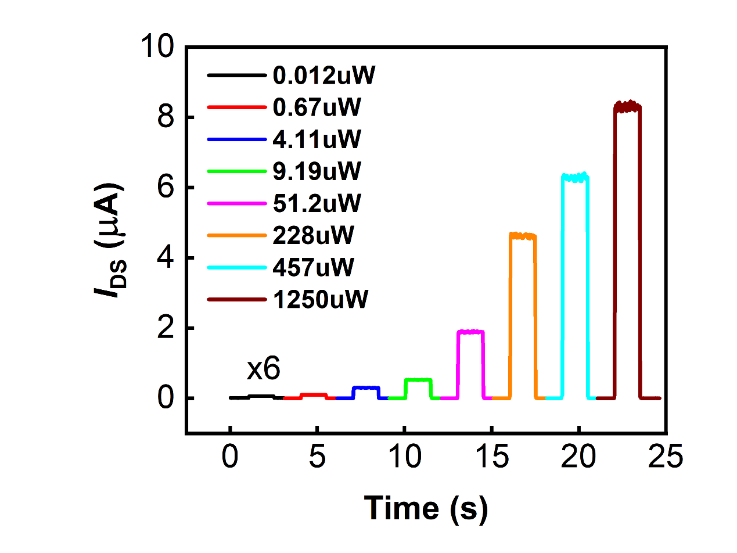


**Figure S7.** The time-resolved photoresponse of our devices under 638 nm illumination at different optical powers.

1. **Optical switching with different gate voltage under a 638 nm laser.**


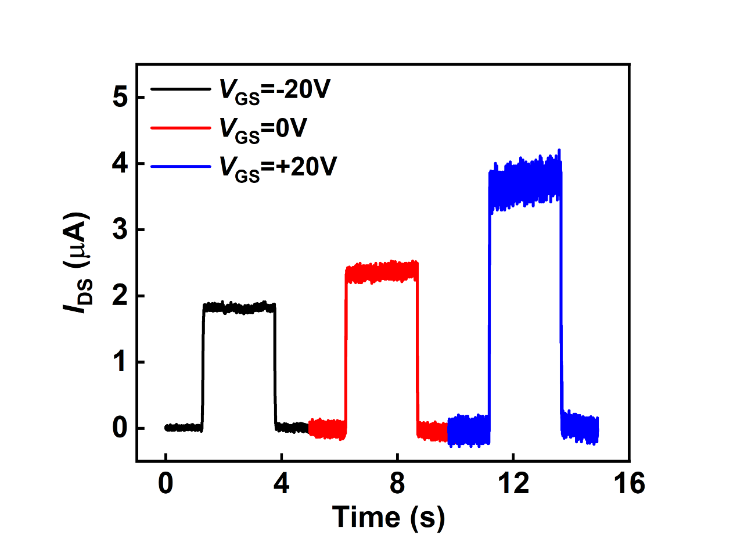


**Figure S8.** The time-resolved photoresponse of our devices under 638 nm illumination at different gate voltage.

1. **The transfer curves of undoped MoS_2_ device.**

The transfer curves of the undoped device under different powers are obtained at *V*_DS_=2V. The device exhibits typical n-type behavior. Its photocurrent shows less variation with different powers than that of doped device, which can be attributed to the PN junctions formed in both the horizontal and vertical directions in the doped device. The calculated optimal responsivity is about 1.38×10^4^ A·W^-1^, which is significantly lower than that of the doped device. Additionally, the undoped device exhibits no photonic response at 1550 nm.

**
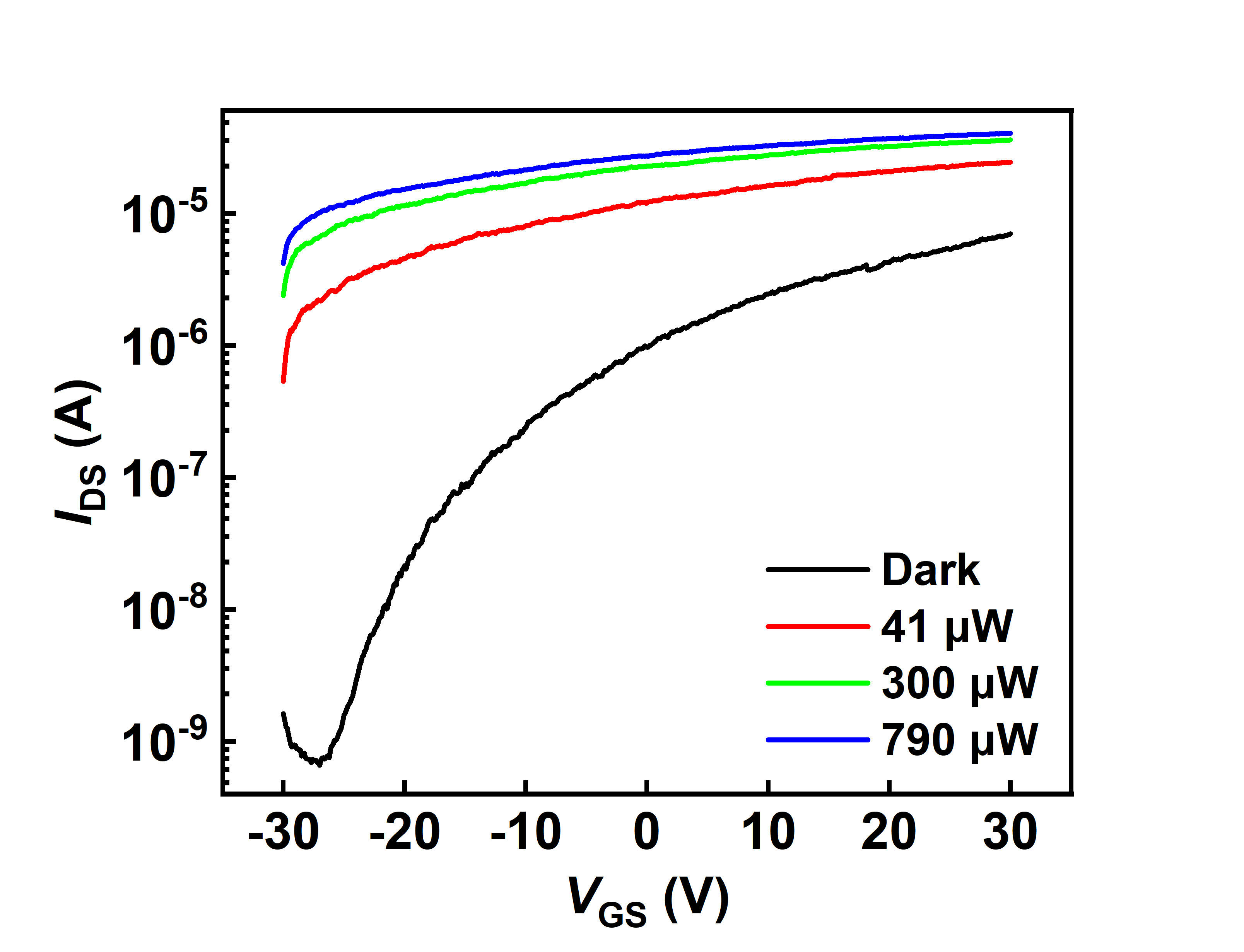
**

**Figure S9.** The transfer curves of undoped MoS_2_ device under different powers at *V*_DS_=2V.

1. **Extracted power dependence photoresponsivity in the visible light.**


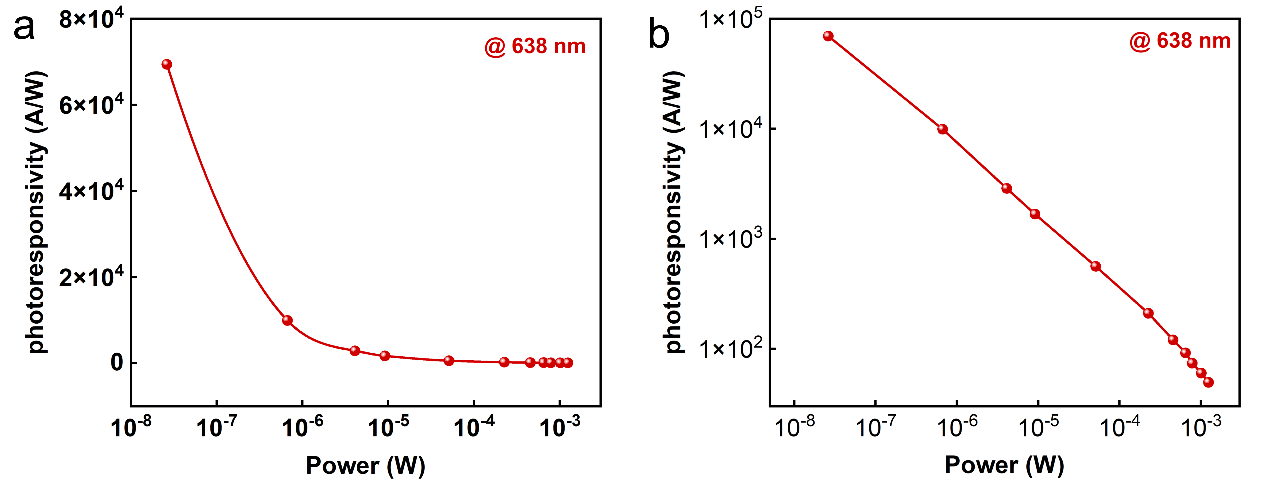


**Figure S10.** The Extracted power dependence photoresponsivity. (a) Linear form. (b) Logarithmic form.

1. **The power-dependent output curves of the undoped device.**

We measured the power-dependent output curves of the undoped device at *V*_GS_=30V. The variation in photocurrent with power is relatively small. The optimal responsivity, approximately 9.67×10^3^ A·W^-1^, is achieved at *V*_DS_=2V, which is more than 7 times lower than that of the doped device at *V*_GS_=25V.

**
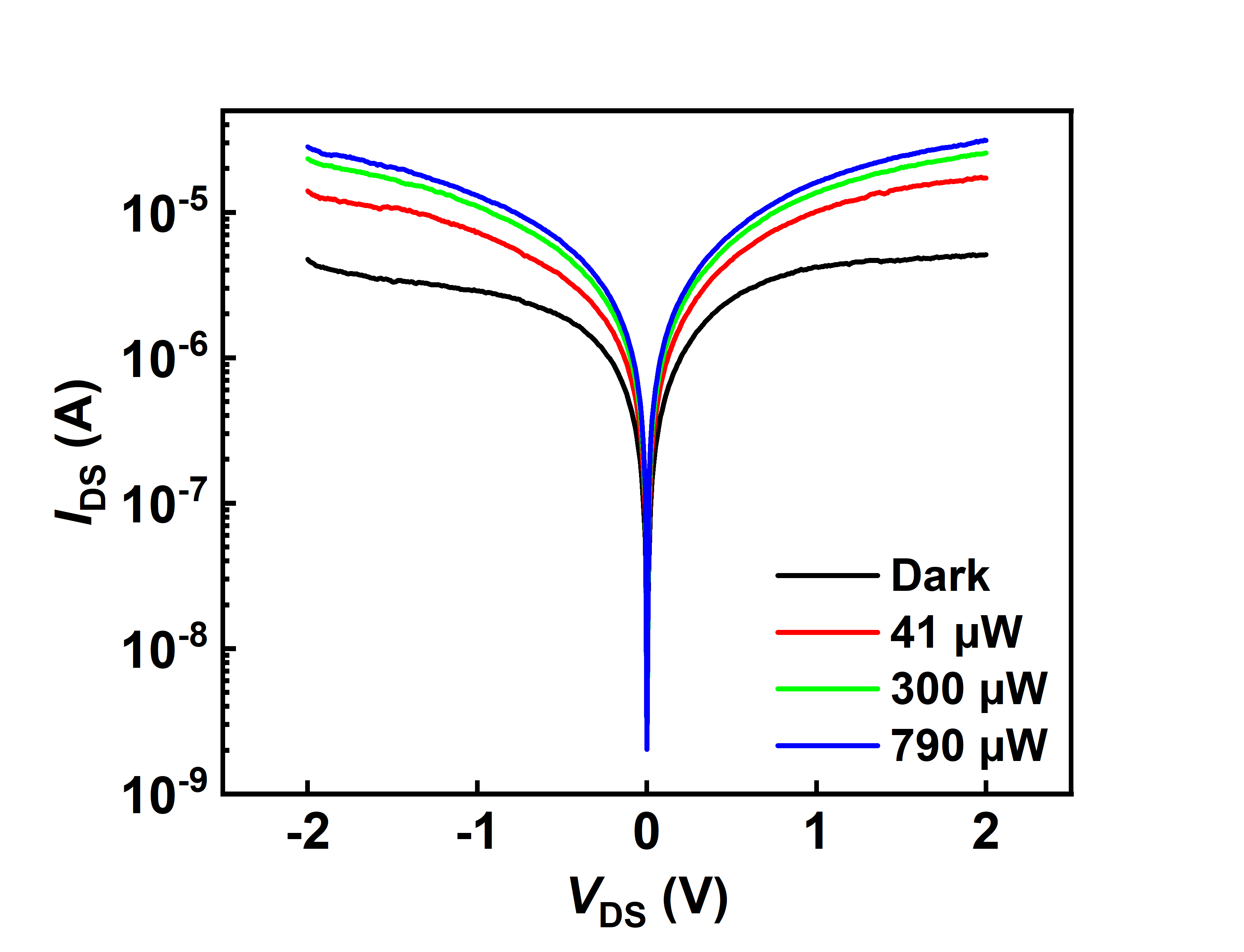
**

**Figure S11.** The power-dependent output curves of the undoped device.

1. **Extracted power dependence photoresponsivity in the shortwave region.**


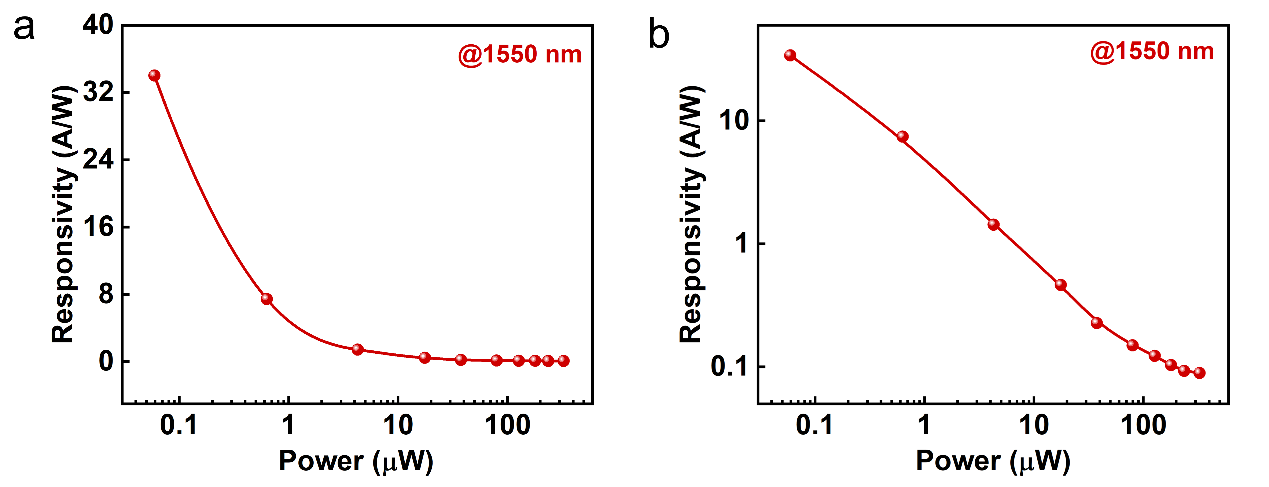


**Figure S12.** The Extracted power dependence photoresponsivity. (a) Linear form. (b) Logarithmic form.

1. **Response time under 638 nm laser illumination at *V*_GS_  =  20 V and *V*_DS_  =  2.0 V.**

**
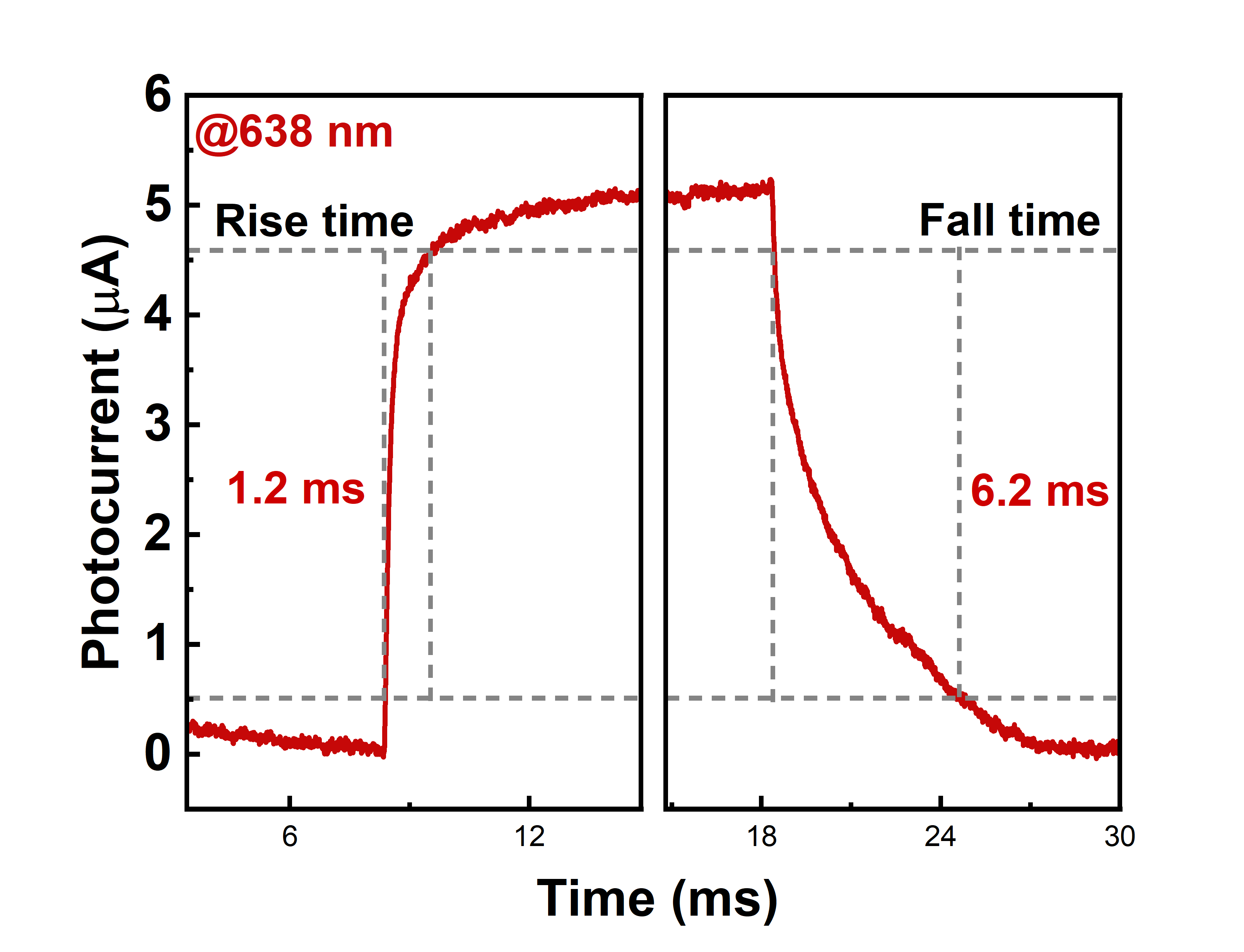
**

**Figure S13.** Response time under 638 nm laser illumination at *V*_GS_  =  20 V and *V*_DS_  =  2.0 V

1. **Relative response in the visible region.**


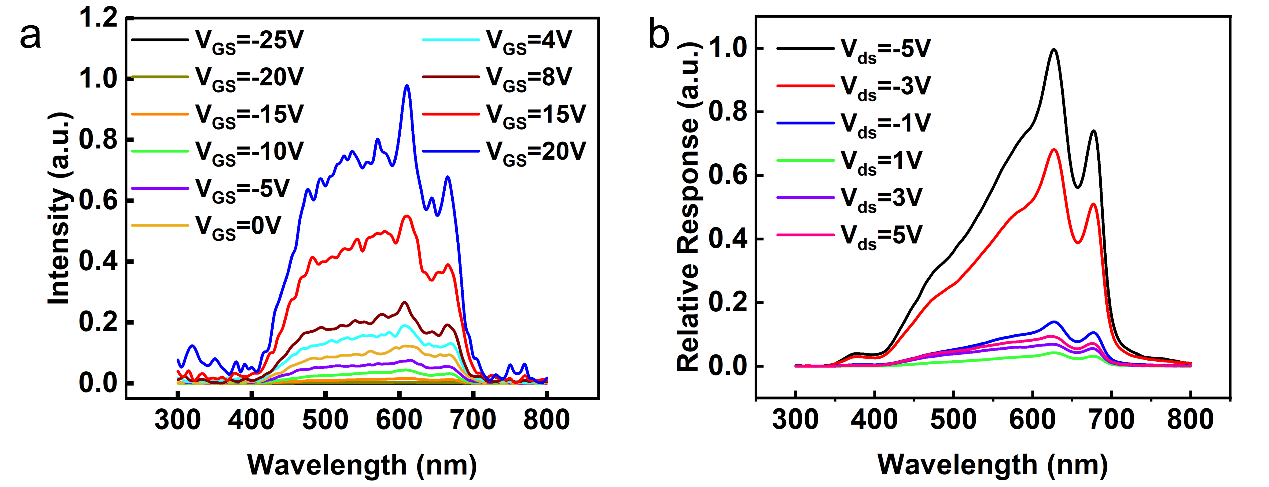


**Figure S14.** The relative responsivity with different bias and gate voltage in the visible region. (a) The relative responsivity with different gate voltage. (b) The relative responsivity with different bias.

1. **Stability of a doped MoS_2_ photodetector.**


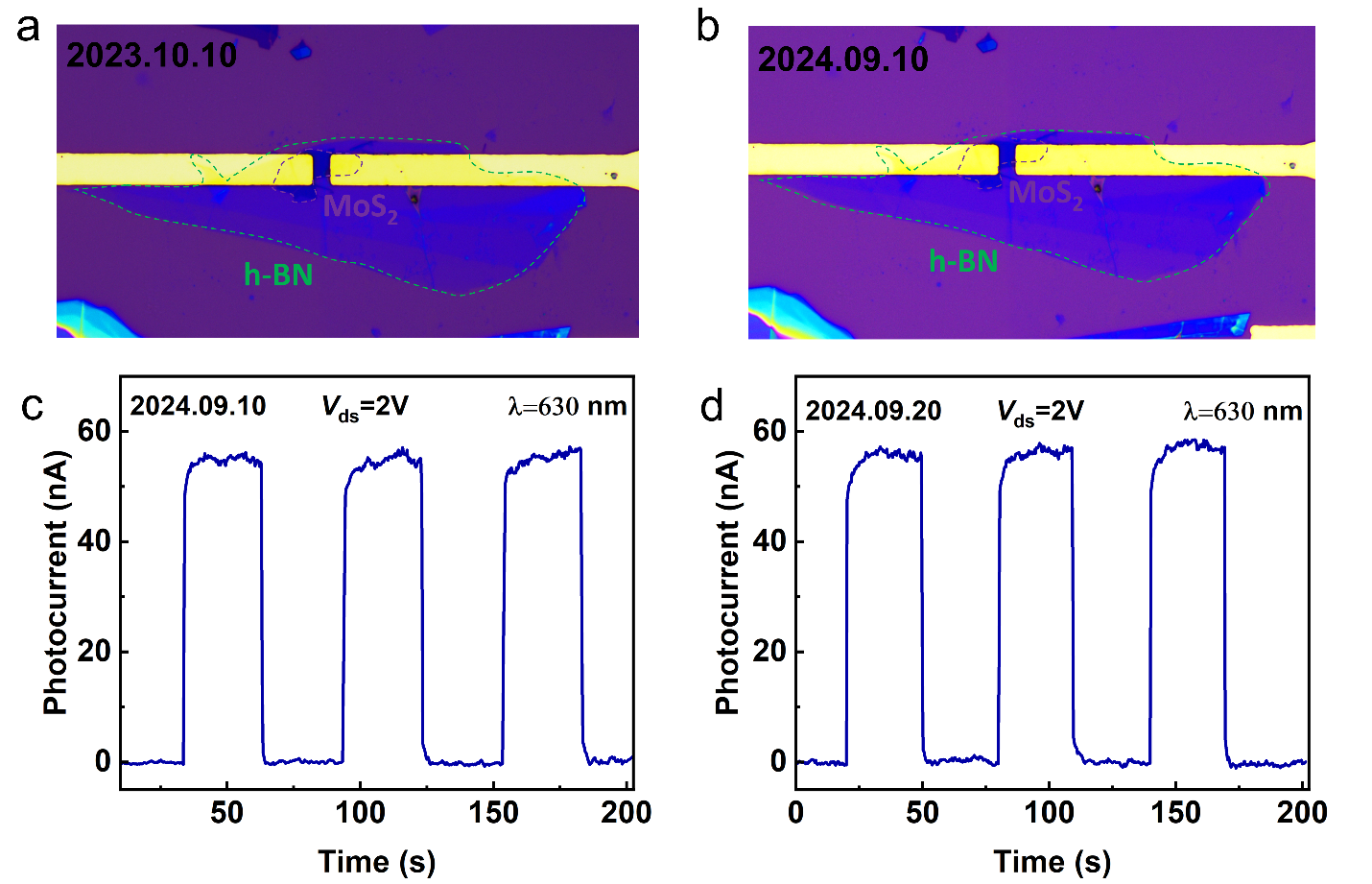


**Figure S15.** Optical images and temporal photoresponse of a doped MoS_2_ photodetector. Optical images of a doped MoS_2_ photodetector protected by h-BN: (a) as fabricated and (b) after being stored in a drybox filled with air for eleven months. Temporal photoresponse of a doped MoS_2_ photodetector: (c) after eleven months and (d) over the subsequent 10 days in the same drybox.

**Table S1. Comparison of the relevant optoelectronic parameters for different 2D MoS_2_-based photodetectors on the visible light.**

| Devices | Wavelength [nm] | R [A·W^-1^] | D* [Jones] | Response Time | Ref. |
| --- | --- | --- | --- | --- | --- |
| PdSe_2_/MoS_2_ JFET | 532 | 600 | 10^11^ | 100/37 ms | ^[9]^ |
| MoS_2_ phototransistors  using back HfO_2_ dielectrics | 460 | 1.1×10^6^ | 5.6×10^13^ | 408/682 ms | ^[10]^ |
|  | 630 | 1.3×10^4^ | - | - |  |
|  | 850 | 13.2 | - | - |  |
| 1-2L MoS_2_ FET using top HfO_2_ dielectrics | 635 | 1.0×10^4^ | 7.7×10^11^ | 10 ms | ^[11]^ |
| MoS_2_ photodetectors with schottky contacts | 650 | 4.1×10^3^ | 1.34×10^13^ | - | ^[12]^ |
|  | 520 | 3.35×10^2^ | 1.27×10^13^ | - |  |
|  | 405 | 0.8×10^2^ | 3.03×10^12^ | - |  |
| Nb-doped WS_2_−MoS_2_ photodiode | 638 | - | 1.1×10^14^ | - | ^[13]^ |
| Ag nanodisks arrays on monolayer MoS_2_ phototransistor | 620 | 2.7×10^4^ | 1.3×10^12^ | 70/410 ms | ^[14]^ |
| WSe_2_−MoS_2_ van der Waals Heterojunction | 532 | 2700 | 5×10^11^ | 17 ms | ^[15]^ |
| MoS_2_ nanosheet Photodetectors by megasonic exfoliation | 515.6 | 10^3^ | 1.8× 10^7^ | 1~2/1 ms | ^[16]^ |
| PtS/MoS_2_ p–n junctions | 400 | 25.43 | 8.54 × 10^12^ | 0.17/0.27 s | ^[17]^ |
|  | 500 | ~11 | ~2 × 10^11^ | ~0.3/0.4 s |  |
|  | 700 | ~5 | ~8 × 10^10^ | 0.65/1.2 s |  |
| MoS_2_ Photodetectors with APTES coating | 655 | 56.5 | 4.47× 10^9^ | - | ^[18]^ |
| Nb-MoS_2_/SnSe_2_ Heterostructure | 254 | 0.5 | 3.3× 10^9^ | 150/234 μs | ^[19]^ |
| MoS_2_ Photodetectors with N-DMBI coating | 530 | 738.2 | 1.3× 10^11^ | 66/144 s | ^[20]^ |
| Nb-MoS_2_ /MoS_2_ homojunction | 550 | 50.4 | 3.0× 10^12^ | 3.9/12.0 s | ^[21]^ |
| vanadium-doped MoS^2^ | 365 | 978.1 | 1.06× 10^12^ | 137/154 ms | ^[22]^ |
| Nitrogen-doped surrounding MoS_2_ homojunction | 638 | 6.94×10^4^ | 1.21 × 10^14^ | 39.7/72.3 μs | This work |

**Table S2. Comparison of the relevant optoelectronic parameters for different 2D MoS_2_-based photodetectors and photodetectors based on other materials for SWIR region.**

| Devices | Wavelength [nm] | R [A·W^-1^] | D* [jones] | Response Time | Ref. |
| --- | --- | --- | --- | --- | --- |
| Cd_3_As_2_/MoS_2_ heterojunction | 1550 | <5 | - | - | ^[23]^ |
| MoS_2_/Ge Heterostructure JFET | 1550 | ~0.1 | 4.43×10^6^ | 40/40 μs | ^[24]^ |
| MoS_2_/Ge Heterostructure | 1550 | 0.4 | 1.3×10^10^ | >3 s | ^[25]^ |
| MoS_2_/Black Phosphorus Heterojunction | 1550 | 0.153 | 2.13×10^9^ | 15/70 μs | ^[26]^ |
| plasmonic-enhanced Ag NPs-MoS_2_ photodetector | 1550 | 0.539 | 0.94×10^9^ | - | ^[27]^ |
| MoS_2_ photodetector integrated with microring resonator | 1516 | 0.155 | - | 9/11 μs | ^[28]^ |
| MoS_2_/Au heterostructures using interface  hybridization | 1550 | 0.017 | 8.5×10^7^ | 144/150 μs | ^[29]^ |
| graphene-MoS_2_-WS_2_ heterostructure | 1550 | 17.1 | - | ~10 ms | ^[30]^ |
| black phosphorus-on-WSe_2_ photogate vertical heterostructure | 1550 | 0.5 | 10^10^ | 800/800 μs | ^[31]^ |
| graphene-based photodetector | 1550 | 83 | 10^8^ | 0.6 μs | ^[32]^ |
| MoS_2_ Photodetector by regulating the composition ratio | 1418 | 0.03 | 1.0×10^9^ | - | ^[33]^ |
| black phosphorus  photodetector | 1550 | 0.65 | - | - | ^[34]^ |
| 2D Bi_2_Se_3_ FET | 1456 | 2.7 | 3.3×10^10^ | 540/470 ms | ^[35]^ |
| 2D Bi_2_O_2_Se photodetector | 1200 | 65 | 3×10^9^ | 1s | ^[36]^ |
| Nitrogen-doped surrounding MoS_2_ homojunction | 1550 | 34 | 5.92 × 10^10^ | 0.98/2.71 ms | This work |

**References：**

[1] C. Hu, C. Yuan, A. Hong, M. Guo, T. Yu, X. Luo, *Appl. Phys. Lett.* **2018**, 113, 041602.

[2] M. Chen, C. Hu, X. Luo, A. Hong, T. Yu, C. Yuan, *Appl. Phys. Lett.* **2020**, 116, 073102.

[3] A. Azcatl, X. Qin, A. Prakash, C. Zhang, L. Cheng, Q. Wang, N. Lu, M. J. Kim, J. Kim, K. Cho, R. Addou, C. L. Hinkle, J. Appenzeller, R. M. Wallace, *Nano Lett.* **2016**, 16, 5437-5443.

[4] W. Zhou, D. Hou, Y. Sang, S. Yao, J. Zhou, G. Li, L. Li, H. Liu, S. Chen, *J. Mater. Chem. A* **2014**, 2, 11358.

[5] Y. Huang, W. Zhou, W. Kong, L. Chen, X. Lu, H. Cai, Y, Yuan, L. Zhao, Y. Jiang, H. Li, L. Wang, L. Wang, H. Wang, J. Zhang, J. Gu, Z. Fan, *Adv. Sci.* **2022**, 9, 2204949.

[6] J. Mao, P. Liu, C. Du, D. Liang, J. Yan, W. Song, *J. Mater. Chem. A* **2019**, 7, 8785.

[7] Z. Du, S. Wang, R. Hu, D. Zhang, J. Gu, X. Chen, J. Shang, B. Li, S. Yang, L. Guo, *Mater. Today* **2021**, 51, 136.

[8] J. Jiang, Y. Zhang, Y. An, L. Wu, Q. Zhu, H. Dou, X. Zhang, *Small Methods* **2019**, 3, 1900081.

[9] H. Wang, Z. Li, D. Li, X. Xu, P. Chen, L. Pi, X. Zhou, T. Zhai, *Adv. Funct. Mater.* **2021**, 31, 2106105.

[10] R. Nur, T. Tsuchiya, K. Toprasertpong, K. Terabe, S. Takagi, M. Takenaka, *Commun. Mater.* **2020**, 1, 103.

[11] D. Kufer, G. Konstantatos, *Nano Lett.* **2015**, 15, 7307-7313.

[12] Y. Sun, L. Jiang, Z. Wang, Z. Hou, L. Dai, Y. Wang, J. Zhao, Y. Xie, L. Zhao, Z. Jiang, W. Ren, G. Niu, *ACS Nano* **2022**, 16, 20272-20280.

[13] V. T. Vu, T. L. Phan, T. T. H. Vu, M. H. Park, V. D. Do, V. Q. Bui, K. Kim, Y. H. Lee, W. J. Yu, *ACS Nano* **2022**, 16, 12073-12082.

[14] H. Y. Lan, Y. H. Hsieh, Z. Y. Chiao, D. Jariwala, M. H. Shih, T. J. Yen, O. Hess, Y. J. Lu, *Nano Lett.* **2021**, 21, 3083-3091.

[15] G. H. Shin, C. Park, K. J. Lee, H. J. Jin, S. Y Choi, *Nano Lett.* **2020**, 20, 5741-5748.

[16] L. Kuo, V. K. Sangwan, S. V. Rangnekar, T. C. Chu, D. Lam, Z. Zhu, L. J. Richter, R. Li, B. M. Szydłowska, J. R. Downing, B. J. Luijten, L. J. Lauhon, M. C. Hersam, *Adv. Mater.* **2022**, 34, 2203772.

[17] F. Li, R. Tao, B. Cao, L. Yang, Z. Wang, *Adv. Funct. Mater.* **2021**, 31, 2104367.

[18] D. H. Kang, M. S. Kim, J. Shim, J. Jeon, H. Y. Park, W. S. Jung, H. Y. Yu, C. H. Pang, S. Lee, J. H. Park, *Adv. Funct. Mater.* **2015**, 25, 4219-4227.

[19] Y. Yu, T. Shen, H. Long, M. Zhong, K. Xin, Z. Zhou, X. Wang, Y. Y. Liu, H. Wakabayashi, L. Liu, J. Yang, Z. Wei, H. X. Deng, *Adv. Mater.* **2022**, 34, 2206486.

[20] S. Qi, W. Zhang, X. Wang, Y. Ding, Y. Zhang, J. Qiu, T. Lei, R. Long, N. Liu, *Nano Res.* **2022**, 15, 9866-9874.

[21] R. Tao, X. Qu, Z. Wang, F. Li, L. Yang, J. Li, D. Wang, K. Zheng, M. Dong, *J. Mater. Sci. Technol.* **2022**, 119, 61-68.

[22] M. Suleman, S. Lee, M. Kim, M. Riaz, Z. Abbas, H. M. Park, V. H. Nguyen, N. Nasir, S. Kumar, J. Jung, Y. Seo, *Mater. Today Phys.* **2024**, 43, 101427.

[23] Z. Huang, Y. Jiang, Q. Han, M. Yang, J. Han, F. Wang, M. Luo, Q. Li, H. Zhu, X. Liu, *Nanotechnology* **2019**, 31, 064001.

[24] B. Wang, L. Wang, Y. Zhang, M. Yang, D. Lin, N. Zhang, Z. Jiang, M. Liu, Z. Zhu, H. Hu, *Adv. Funct. Mater.* **2022**, 32, 2110181.

[25] Y. Zhang, B. Wang, Z. Han, X. Shi, N. Zhang, T. Miao, D. Lin, Z. Jiang, M. Liu, H. Guo, J. Zhang, H. Hu, L. Wang, *ACS Photonics* **2023**, 10, 1575-1582.

[26] L. Ye, H. Li, Z. Chen, J. Xu, *Acs Photonics* **2016**, 3, 692-699.

[27] M. J. Park, K. Park, H. Ko, *Appl. Surf. Sci.* **2018**, 448, 64-70.

[28] Q. Zhang, Y. Ji, S. Hu, Z. Li, C. Li, L. Gu, R. Tian, J. Zhang, L. Fang, B. Zhao, J. Zhao, X. Gan, *Appl. Phys. Lett.* **2022**, 120, 261111.

[29] C. Hong, S. Oh, V. K. Dat, S. Pak, S. Cha, K. H. Ko, G. M. Choi, T. Low, S. H. Oh, J. H. Kim, *Light: Sci. Appl.* **2023**, 12, 280.

[30] Y. F. Xiong, J. H. Chen, Y. Q. Lu, F. Xu, *Adv. Electron. Mater.* **2019**, 5, 1800562.

[31] L. Ye, P. Wang, W. Luo, F. Gong, L. Liao, T. Liu, L. Tong, J. Zang, J. Xu, W. Hu, *Nano Energy* **2017**, 37, 53-60.

[32] Z. Chen, X. Li, J. Wang, L. Tao, M. Long, S. J. Liang, L. K. Ang, C. Shu, H. K. Tsang, J. B. Xu, *ACS nano* **2017**, 11, 430-437.

[33] Y. Xie, B. Zhang, S. Wang, D. Wang, A. Wang, Z. Wang, H. Yu, H. Zhang, Y. Chen, M. Zhao, B. Huang, L. Mei, J. Wang, *Adv. Mater.* **2017**, 29, 1605972

[34] N. Youngblood, C. Chen, S. J. Koester, M. Li, *Nat. Photonics* **2015**, 9, 247-252.

[35] F. Wang, L. Li, W. Huang, L. Li, B. Jin, H. Li, T. Zhai, *Adv. Funct. Mater.* **2018**, 28, 1802707.

[36] J. Yin, Z. Tan, H. Hong, J. Wu, H. Yuan, Y. Liu, C. Chen, C. Tan, F. Yao, T. Li, Y. Chen, Z. Liu, K. Liu, H. Peng, *Nat. Commun.* **2018**, 9, 3311.
